# Physics-based nucleosome-resolution modeling of epigenetic-driven chromatin domain dynamics

**DOI:** 10.1093/nar/gkag535

**Published:** 2026-05-26

**Authors:** Chenyang Gu, Shoji Takada, Giovanni B Brandani

**Affiliations:** Department of Biophysics, Graduate School of Science, Kyoto University, Kyoto 606-8502, Japan; Department of Biophysics, Graduate School of Science, Kyoto University, Kyoto 606-8502, Japan; Department of Biophysics, Graduate School of Science, Kyoto University, Kyoto 606-8502, Japan

## Abstract

Chromatin spatially organizes the eukaryotic genome to support key cellular processes such as gene regulation, but the interplay between epigenetics, chromatin structure, and function is still poorly understood. We propose a nucleosome-resolution coarse-grained model that captures the essential features of chromatin organization over multiple scales: from nucleosome dynamics to chromatin fiber folding, and liquid–liquid phase separation. The model describes the effects of DNA linker length, histone tail acetylation, linker histone H1, and multibromodomain proteins such as BRD4. It is designed to be experimentally accurate but computationally efficient, allowing the study of 100 kb genomic regions on a timescale of seconds with moderate resources. We apply this model to explore the structure and dynamics of two active loci of mouse embryonic stem cells, Pou5f1 and Sox2, as determined solely by epigenetics. Our simulations reveal that chromatin folds into liquid-like domains characterized by similar histone modifications. These domains are highly dynamic, driving the formation of transient contacts between distant *cis-*regulatory regions. *In silico* mutation studies further clarify the roles of individual epigenetic factors. Overall, our physics-based modeling establishes that epigenetic-dependent nucleosome interactions play a key role in shaping the functional organization of genomic loci.

## Graphical Abstract

**Graphical Abstract:**
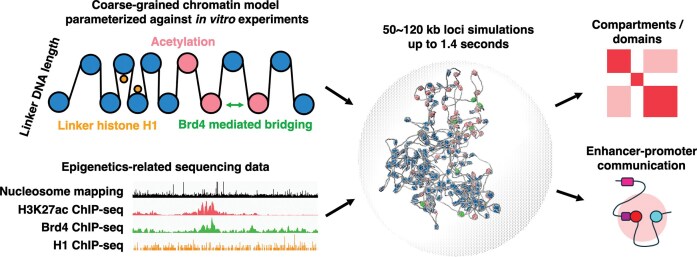

## Introduction

The hierarchical spatial organization of the eukaryotic genome is critical for its function. Genomic DNA assembles with histone octamers to form nucleosomes, the basic structural unit of chromatin (∼10 nm). Nucleosomes interact to form higher order structures including nucleosome clutches (∼20 nm) [1, 2], packing domains (PDs, 50∼200 nm) [3, 4], topologically associating domains (TADs) [5], and chromatin compartments [6]. These domain structures are believed to aid the regulation of gene expression by controlling the assembly of the transcription machinery, bringing distant enhancers and promoters into proximity, or insulating genomic loci.

The Structural Maintenance of Chromosomes (SMC) complex cohesin has been recognized as a key player in the establishment of TADs through the mechanism of loop extrusion [7, 8]. However, the majority of TADs are maintained upon acute loss of loop extrusion factors [9], suggesting that additional mechanisms likely play a key role in chromatin 3D organization. The 1D pattern of epigenetic marks along the genome, such as histone tail acetylation, is another key factor long suspected to shape the 3D structure of the genome. The first Hi-C experiments already revealed how mammalian genomes are spatially organized into distinct large-scale compartments occupied by either active regions enriched in histone tail modifications (A compartment), or by inactive, gene-poor regions (B compartment) [6]. Recent ultra-deep Hi-C data show that such organization persists down to the sub-TAD scale of individual genes, which are often organized into an alternating patterns of two chromatin compartments with an average interval size of 12.5 kilobases (kb) [10]. The two compartments correlate with euchromatin/heterochromatin markers similarly to the original A/B compartmentalization, but on a smaller scale.

A recent experimental study measuring the condensability of native nucleosomes by next-generation sequencing suggested that chromatin compartments are formed by phase separation through direct nucleosome–nucleosome interactions modulated by epigenetic modifications [11]. This is consistent with the intrinsic tendency of chromatin to form complex epigenetic-dependent liquid condensates *in vitro* [12]. These experiments suggest a regulation mechanism for *in vivo* chromatin domains, involving acetylation, linker histone H1, multibromodomain proteins, and linker DNA length (controlling the spacing between nucleosomes along the genome). Despite this accumulating evidence, the extent to which epigenetics can shape the functional organization of active genomic loci in the absence of loop extrusion is still unclear.

Computer modelling provides suitable tools to address this question, as it allows us to explore the dynamic organization of chromatin in controlled settings with high spatial and temporal resolution. In particular, coarse polymer simulations have been successfully employed to explore the roles of specific physical mechanisms shaping the 3D genome, such as SMC loop extrusion [8, 13], bridging-induced attraction [14, 15], epigenetic-driven phase separation [16–19], and nucleosome positions [20]. Accurate chromatin models have also been achieved by systematic coarse-graining directly from experimental contact frequencies [21] or by integrating such data into prior chromatin models [22–24], but these approaches do not directly address the question of how chromatin organization naturally arises from the physical interactions between its components.

To unravel the role of epigenetics on the 3D organization of genes *in vivo*, we would ideally like to make predictions from the bottom up based on the physics of chromatin. There is a long history of all-atom and near-atomic coarse-grained molecular dynamics (MD) simulations that investigate nucleosomes [25–27], and nucleosome–nucleosome interactions [28, 29]. Coarser, but still physics-based, nucleosome-level models have been successfully applied to explore the 3D structure and dynamics of chromatin [30–34], but sampling over sufficiently long timescales to observe the functional dynamics of genes remains a challenge.

In this work, we propose the Nucleosome Interaction Coarse-Grained (NICG) model of chromatin, a physics-based model optimally designed to explore the epigenetic-dependent phase separation of chromatin into liquid domains. By representing proteins with one bead per histone and DNA with one bead per helix turn, our model has sufficient detail to capture the fundamental features of epigenetic-dependent nucleosome–nucleosome interactions, but efficient enough to explore the dynamics of large (∼100 kb) genomic loci over the timescale of seconds, allowing us to observe large-scale functional rearrangements of chromatin domains.

We applied our model to study the Pou5f1 and Sox2 loci of mouse embryonic stem cells (mESCs). We build a model of the target loci by incorporating key experimental epigenetic patterns that could potentially influence the phase separation of chromatin: nucleosome positions from chemical mapping [35], H3K27 acetylation ChIP-seq [36], histone H1 ChIP-seq [37], and multibromodomain protein BRD4 ChIP-seq [38]. Our large-scale simulations reveal that the folding of the target loci is controlled by the underlying epigenetic profiles, with distinct roles played by acetylation, H1, and BRD4. These domains are highly dynamic and support the formation of direct contacts between distant enhancers and promoters, showing direct evidence for a role of epigenetics in the functional organization of our genome.

## Materials and methods

### Nucleosome interaction coarse-grained model of chromatin

**Figure 1.**
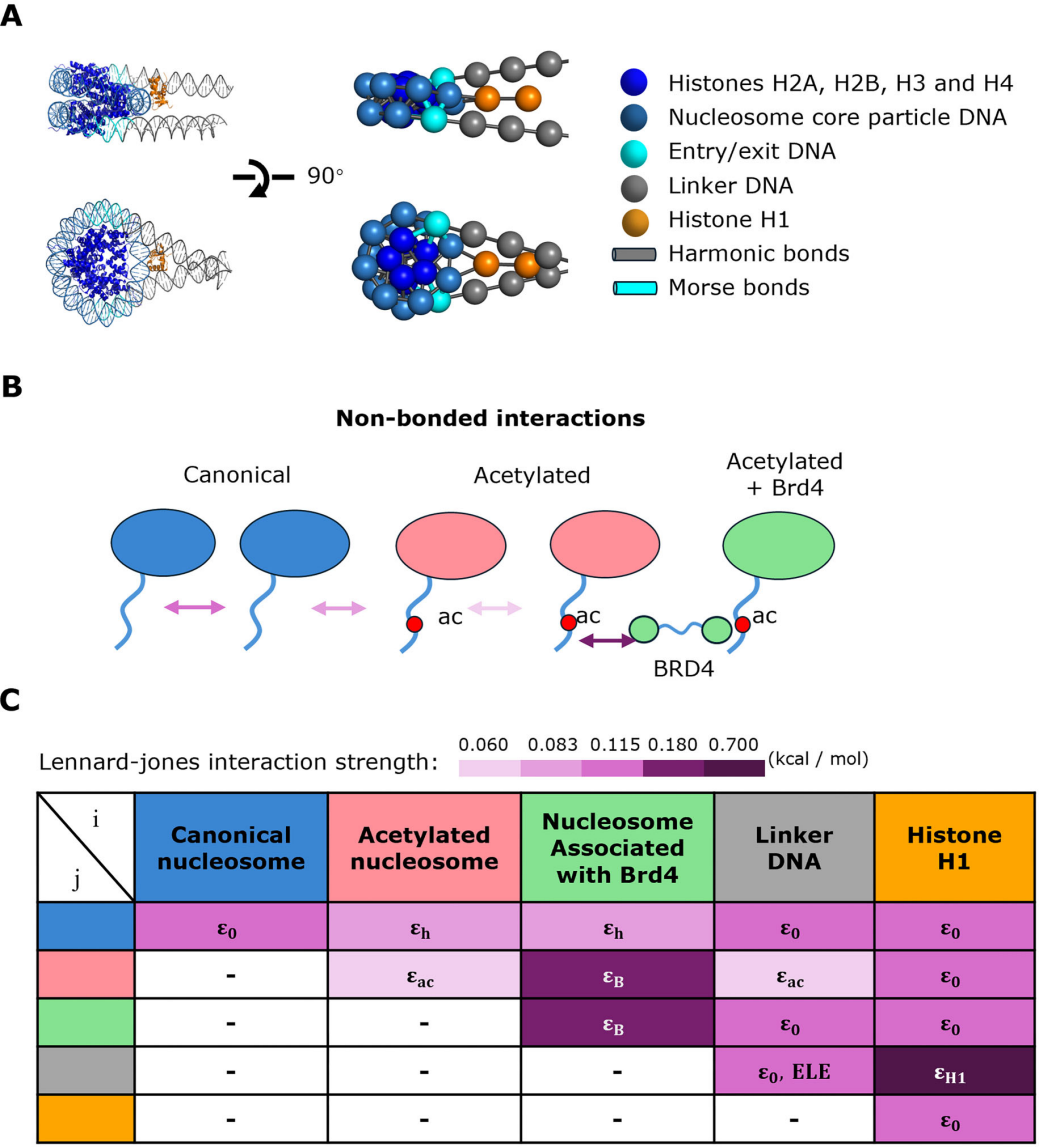
Architecture of coarse-grained chromatin model. (**A**) Comparison between the cryo-electron microscopy (cryo-EM) structure (PDB ID: 7K5Y) and the coarse-grained model of a chromatosome. In our model, we use one bead for each H2A, H2B, H3, or H4 histones, two beads for linker histone H1 (one for the globular part, and one for the disordered C-terminal tail, which is not visible in the cryo-EM structure), and one bead for every DNA helical turn (10.5 bp). (**B**) Schematics of nonbonded interactions between nucleosome beads and the mechanism of BRD4 bridging acetylated nucleosomes. (**C**) Table of the nonbonded interaction strengths between the five groups of beads in our model. ε_0_, ε_h_, ε_ac_, ε_H1_: Lennard–Jones interaction strengths. ELE indicates Debye–Hückel electrostatic interaction. Relative strengths of Lennard–Jones interactions are indicated by color intensity.

In our model, which we call the NICG model, we represent chromatin using solely point particles, with one bead for each histone protein in the nucleosome core, one bead for each B-form DNA helical turn (10.5 bp) [39], and two beads for linker histone H1. Each nucleosome is constructed by arranging the 147 bp of nucleosomal DNA (=14 beads) along a superhelix with a radius of 40 Å and a pitch of 25 Å [40], and the 8 histone beads along a superhelix passing through the same axis and with the same pitch, but with a smaller radius of 18 Å (Fig. 1A). Linker histone H1 is represented by two beads, one for the globular domain and one for the long, disordered C-terminal tail. When present, the H1 beads are positioned along the dyad, with the first bead 70 Å away from the nucleosome center. We employed a total of 20 bead types grouped into 5 classes characterized by the same interactions with others: linker DNA, canonical nucleosome, acetylated nucleosome, acetylated nucleosome associated with BRD4, and linker histone (Supplementary Table S1). All beads have the same size σ = 35 Å, corresponding to the length of a DNA helical turn.

The total potential energy is defined by contributions from local bond and angle potentials, and nonlocal Lennard–Jones and Debye–Hückel electrostatic interactions (Supplementary Table S2). Harmonic bonds are applied between all pairs of beads closer than 38 Å in the reference nucleosome geometry, and between consecutive linker DNA beads (Fig. 1A). The first and last nucleosomal DNA beads (entry/exit DNA) were specially treated, and connected to the neighboring core beads by Morse bonds instead of harmonic bonds (Supplementary Information, Supplementary Fig. S1A), which allows us to model the stochastic unwrapping of DNA from the histones (nucleosome breathing) [41, 42]. We applied angle potentials along the DNA chain to model its bending elasticity. Angle potentials are also applied between the first H1 bead and neighboring nucleosome beads to maintain the H1 globular domain dyad positioning [43]. Our model does not account for DNA torsional elasticity and the effects of torsional stresses, reflecting the effects of Type I DNA topoisomerases [44], and also the accumulation of DNA twist within nucleosome core particles [45]. Nevertheless, it is important to note that torsional stresses may be important in certain contexts [26], such as when studying the response of chromatin structure to gene transcription by RNA polymerase [46].

Nonbonded beads interact via a Lennard–Jones potential with strength varying according to their bead type, as described in Fig. 1B and C. In particular, attraction was weakened for pairs of acetylated nucleosomes, but strengthened again for pairs where at least one acetylated nucleosome is associated with BRD4, to implicitly model the bridging effect of multibromodomain proteins [12, 47] (Fig. 1B). Interactions between linker DNA beads and H1 beads are strengthened to model H1-mediated compaction of nucleosome fibers. Linker DNA beads and H1 beads are also charged (−7.35e and +5e per bead, respectively) and they interact via an additional Debye–Hückel potential. Charge values are optimized to account for effective counter-ion condensation within the considered framework of Debye–Hückel electrostatics.

### Coarse-grained simulation

All simulations were executed using the molecular dynamics software LAMMPS [48]. Temperature was set to 300 K, while, except when noted, monovalent salt concentration was set to 150 mM to reflect physiological conditions. The equations of motion were integrated by Langevin dynamics. A uniform mass of one unit was assigned to all bead types. Based on the analysis of chromatin diffusion (see section “Chromatin forms highly dynamic domains”), a scale factor of 1.4 ns/timestep was considered when comparing simulated time and real timescales. The Langevin damping factor was set at 140 ns, which corresponds to a low viscosity. Coordinates of all particles were saved every 10^5^ timesteps for further analysis.

### Force field parametrization and validation

The force field was parametrized based on a series of experimental data on chromatin organization: DNA persistence length, nucleosome unwrapping, and chromatin fiber sedimentation coefficients. It was then validated on experimental chromatin liquid–liquid phase separation properties.

**Figure 2.**
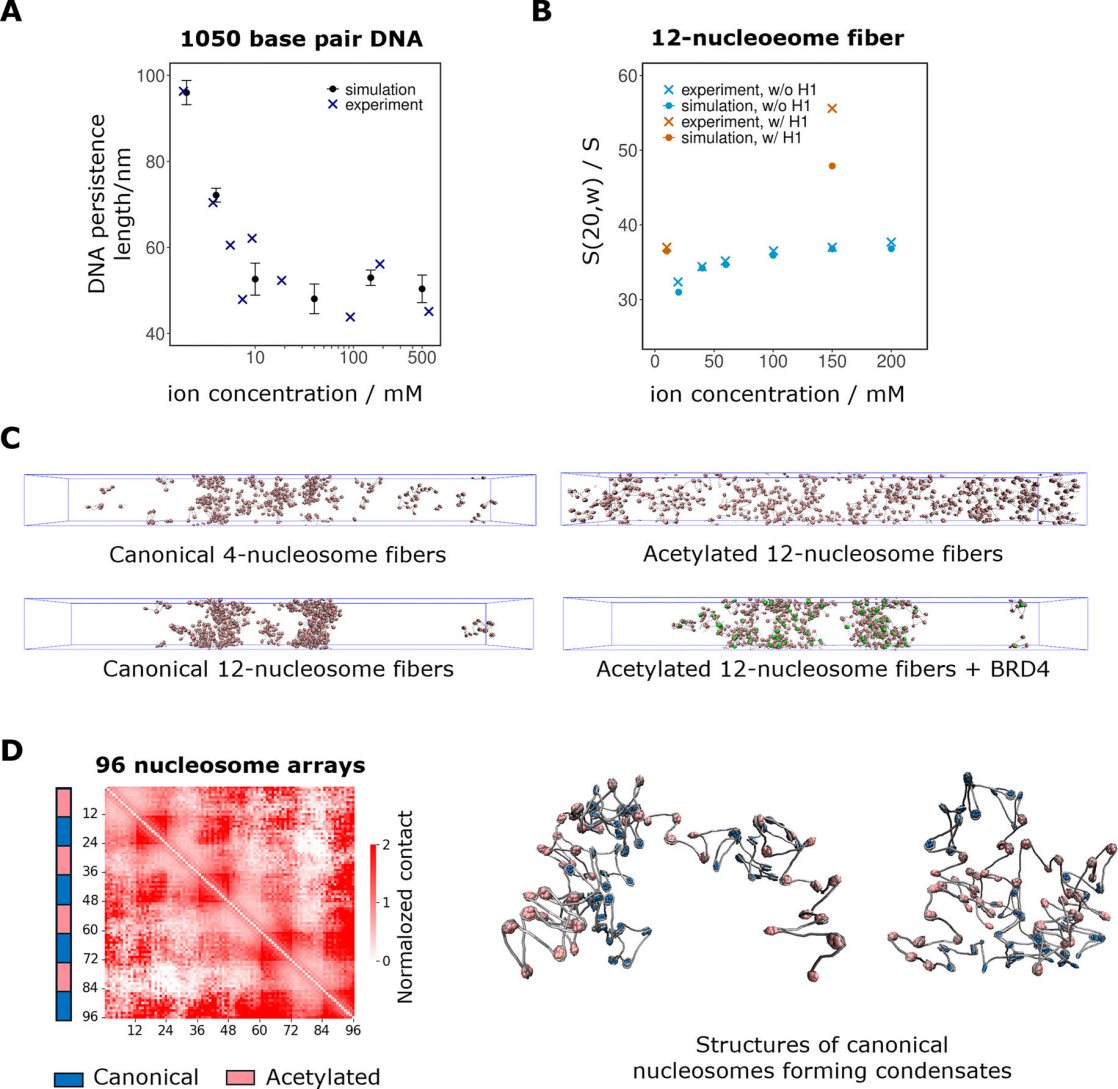
Model parameterization and validation. (**A**) DNA persistence length as a function of salt concentration calculated from 1050 bp DNA simulations, compared with experimental data from Baumann *et al*. [49]. (**B**) Sedimentation coefficient of canonical 12-nucleosome fibers and canonical 12-nucleosome fibers with linker histone H1 as a function of salt concentration calculated from simulations, compared with experimental data from Hansen *et al*. and Grigoryev *et al*. [53, 54]., so this (**C**) Slab simulation snapshots of 90 copies of canonical 4-nucleosome fibers or 45 copies of 12-nucleosome fibers with different modifications at the end of 10^8^ MD timesteps. (**D**) Normalized self-contact frequency map of 96-nucleosome arrays of alternating canonical and acetylated nucleosomes (left), and representative structure snapshots showing the condensation of canonical nucleosomes (right).

First, we simulated a 1050-bp DNA molecule (100 beads) at various salt concentrations to parametrize the elastic constant of the DNA angle potential and the electric charge on naked DNA and linker DNA beads. We performed a large number of simulations to find the optimal set of parameters that matched the experimental dependence of DNA persistence length on ionic strength (Fig. 2A) [49]. Our current coarse-grained model captures the average behavior of DNA, but a sequence-dependent persistence length [50] can be implemented in the future based on its linear dependence on the bending stiffness (confirmed based on additional simulations, see Supplementary Fig. S2) [51]. While sequence-dependent flexibility influences local chromatin architecture [52], our study focuses on large-scale domain formation. At this scale, a uniform persistence length offers a robust approximation for isolating epigenetic-driven mechanisms. Sequence-dependent nucleosome dynamics can be important especially near transcription start sites [52], but its role is outside the current research focus on nucleosome–nucleosome interactions.

To optimize the Morse potential at the nucleosomal DNA entry/exit beads, we simulated two nucleosomes connected by a 63-bp linker DNA. The calculated equilibrium constant of spontaneous nucleosome unwrapping, 0.10 ± 0.01 (see Supplementary Methods), was consistent with the experimental value at physiological conditions reported by Li and Widom (0.02–0.1) [55], but somewhat smaller than the estimate by Koopmans *et al*. (0.2–0.6) [56].

For acetylated nucleosomes, the unwrapping equilibrium constant from simulations is 0.32 ± 0.02, a three-fold increase relative to canonical ones (Supplementary Fig. S1B). Our model represents an “average” acetylated nucleosome with multiple acetylations on histone tails, so this is consistent with experiments showing that individual H3 or H4 acetylations increase the unwrapping probability by about a factor of 2 [57].

Most of the remaining nonbonded interactions (the Lennard–Jones strengths ε, summarized in Fig. 1C, and the H1 charge) are parametrized to reproduce salt-dependent sedimentation coefficients s_20,w_ of chromatin fibers [53, 54]. We performed simulations of 12-nucleosome fibers with 207-bp nucleosome repeat length (NRL) in the presence and absence of linker histone H1, in salt concentrations ranging from 10 mM to 200 mM (Fig. 2B). The measured sedimentation coefficients are in good agreement with experimental data, except for some discrepancy at 150 mM salt in the presence of H1. This behavior is however consistent with the predictions from other coarse-grained models of chromatin [54], and could be due to limitations in the prediction of the sedimentation coefficient from simulations in this regime [58]. Our model also reproduces the experimental increase of sedimentation coefficient (compaction) with decreasing NRL [59] (Supplementary Fig. S3), but not its 10-bp periodicity due to the lack of DNA torsional potential. For acetylated nucleosomes, the Lennard–Jones interaction strength ε_ac_ was reduced to about half of ε_0_ (from 0.115 kcal/mol to 0.06 kcal/mol) to match experiments showing that acetylation reduces the sedimentation coefficients 11–15% under physiological salt concentrations [60, 61]. The hybrid Lennard–Jones interaction strength between one bead from a canonical nucleosome core particle and one bead from an acetylated nucleosome core particle, ε_h_, is set according to the geometric average mixing rule. When present, linker histone beads interact with linker DNA beads via both electrostatic and Lennard–Jones interactions, the latter with enhanced strength ε_H1_. See Supplementary Information section on model parametrization for more details.

We also performed additional simulations (see Supplementary Methods) to estimate the potential of mean force (PMF) along the nucleosome–nucleosome distance in the stacked configuration (Supplementary Fig. S4). The free energy difference between stacked and unbound configurations is about 5 k_B_T for canonical nucleosomes and 2 k_B_T for acetylated nucleosomes. For canonical nucleosomes, our value falls in the middle of the wide range reported from experiments: from 2 k_B_T [62] to 14 k_B_T [63]. Recent simulations using explicit ions suggested that such large deviation depends on the constraints imposed on nucleosome orientation by the experimental setup [64]. Consistent with experimental data [62], histone acetylation reduces the depth of the PMF to less than a half of the canonical value.

We validated our overall model through large-scale simulations of chromatin liquid–liquid phase separation using a slab geometry with a 100 × 100 × 1000 nm^3^ simulation box (Fig. 2C), under conditions where chromatin fibers are experimentally known to condense or not [12]. As a negative control, we placed within the simulation box 90 copies of 4-nucleosome fibers; consistent with experiments, these short fibers did not phase separate in our simulations. We then placed 45 copies of 12-nucleosome fibers in the same box, giving an initial nucleosome concentration of 90 μM. In this case the fibers phase separated into a dilute phase with a concentration of 12 ± 1 μM and a condensed phase with a concentration of 294 ± 12 μM (Supplementary Fig. S5A), which is consistent with the experimental observation of a condensed phase with a concentration of 342 μM. We note some discrepancy with the experimental concentration of the dilute phase (32 nM), indicating that phase separation would occur earlier in experiments. However, both experiments and simulations predict phase separations of unmodified 12-nucleosome fibers starting from concentrations close to that of the nucleus (∼100 μM), which is the regime we are most interested in. When we then placed 45 copies of acetylated 12-nucleosome fibers in the same box, we did not observe phase separation, consistent with experiments. Finally, we placed in the box 45 copies of acetylated 12-nucleosome fibers, where on each fiber 2 nucleosomes are also associated with BRD4. In this simulation, acetylated nucleosomes associated with BRD4 interact with other acetylated nucleosomes with enhanced Lennard–Jones strength ε_B_ (whether these are bound to BRD4 or not). In agreement with experiments, BRD4 association recovers the condensation of acetylated fibers (Fig. 2C), confirmed by a higher number of nucleosome–nucleosome contacts (Supplementary Fig. S5B).

As a final validation, we simulated a 96-nucleosome array alternating between canonical and acetylated every 12 nucleosomes (Fig. 2D), as investigated in recent *in vitro* experiments [65]. The resulting contact maps show a checkerboard-like pattern similar to those of experiments, with high contact frequencies between pairs of canonical nucleosomes, and lower contact frequencies between acetylated and other nucleosomes (Fig. 2D, left panel).

### Pou5f1 and Sox2 loci model

**Figure 3.**
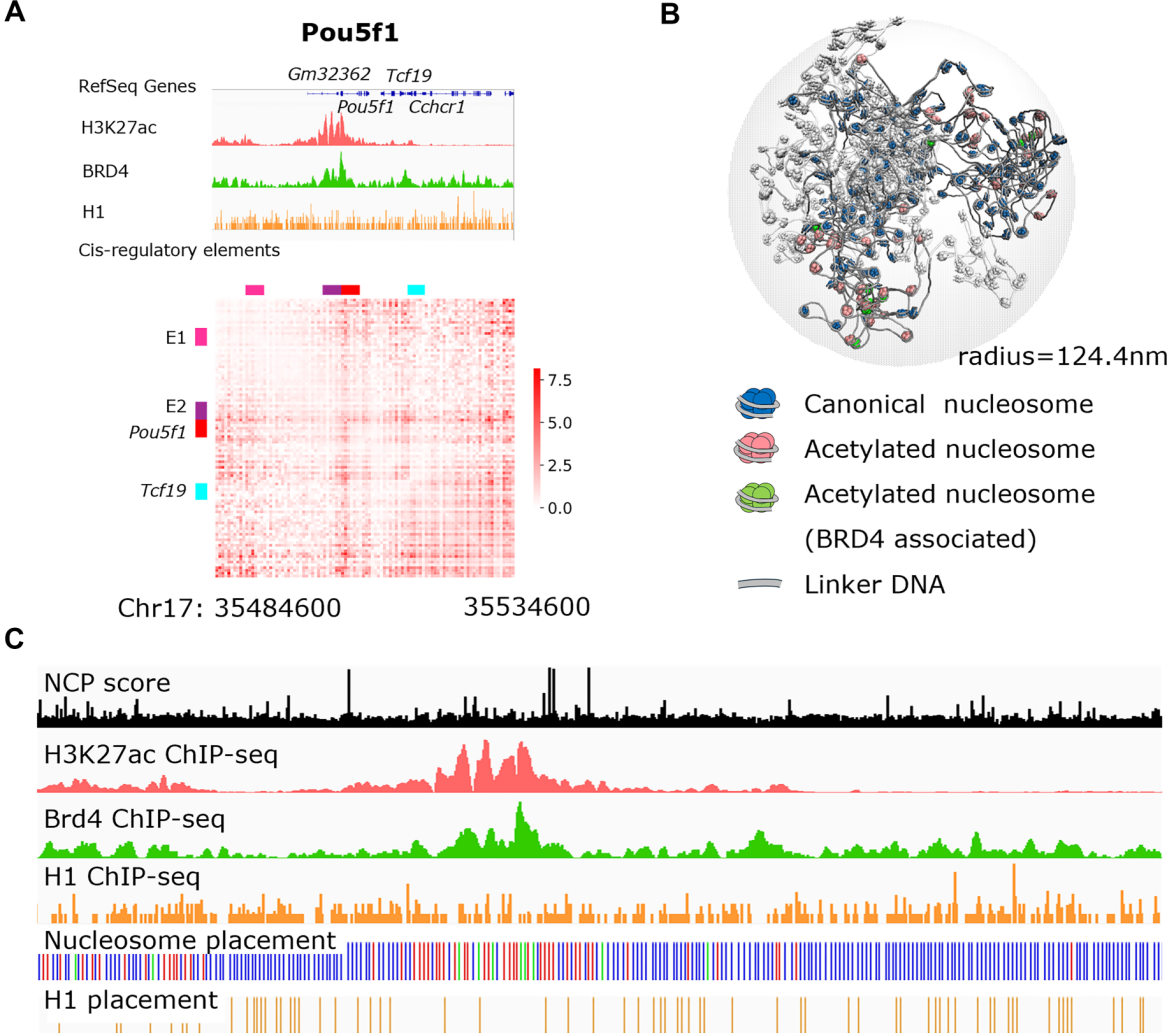
Coarse-grained modeling of the Pou5f1 locus. (**A**) Sequencing data of nucleosome interaction regulatory factors (H3K27ac, BRD4, and H1) and normalized Micro-C contact frequency map of the target genomic locus [69]. (**B**) Initial structure of the Pou5f1 locus after a short relaxation by MD. Canonical, acetylated, and BRD4-associated nucleosomes in blue, pink, and green, respectively. The short chromatin fibers that are not the target locus are represented in white. (**C**) Mapping of nucleosome positions and modifications according to sequencing data [nucleosome colors as in panel (B)].

The initial structures for the simulations of the Pou5f1 and Sox2 loci of mESCs were prepared based on experimental information on nucleosome positions from chemical mapping (GSM2183909) [35], and epigenetic profiles from ChIP-seq data (Fig. 3 and Supplementary Information methods). Acetylated nucleosomes are assigned according to the H3K27ac ChIP-seq profile in mESCs (GSM3399478) [36]. Linker histone H1 is added to nucleosome dyad sites according to their H1 ChIP-seq profile (GSM1199586). BRD4 is assigned to acetylated nucleosomes according to the BRD4 ChIP-seq profile (GSM2319260) [38]. Nucleosome positions were determined by peak calling based on the chemical mapping scores, resulting in an average NRL of 195 bp, which is larger than the peak in the distribution of genomic distance between neighboring nucleosomes [35]. While ChIP-seq signals represent cell population averages, we utilized them to define probability distributions for nucleosome states. Our model therefore constructs a stochastic realization of the chromatin fiber sampled from these distributions. This results in a discrete fiber that serves as a mechanistic model for a single cell possessing these specific epigenetic patterns. See Supplementary Information section “Mapping of Epigenetic Patterns” for more details.

To recreate the chromatin density of mESCs nuclei, the spherical simulation space is filled with five short (10–16 kb) chromatin fibers in addition to the target locus, so that the average DNA concentration in the simulation space is 12.4 Mbp/μm^3^ (giving a nucleosome concentration of ∼100 μM), as expected assuming the same DNA concentration as that in a mouse (5.4 Gbp) cell nucleus with a radius of 4.7 μm [66]. Compared to using periodic boundary conditions in a cubic box, the spherical walls prevent unrealistic chromatin contacts across the boundary, and the additional chromatin fibers allow the spherical regions to be large enough to keep constraints from the walls to a minimum. Under the assumption that the chromatin environment surrounding these active euchromatin loci are euchromatin regions of similar composition as the considered loci, we assign 20% of the nucleosomes as acetylated, 31% of the nucleosomes as H1-bound, and 16.7% of the acetylated nucleosomes as BRD4-bound (the average values estimated from the number of canonical, acetylated, and BRD4-bound nucleosomes in the Pou5f1 and Sox2 loci).

The initial structures of the respective loci were generated by growing chromatin fibers that follow random reference backbones within spheres of radius 124.4 nm for Pou5f1 and radius 156.7 nm for Sox2 (more details in Supplementary Information and Supplementary Fig. S6). Before the production runs, the initial structures, which may contain steric clashes, were first relaxed by molecular dynamics simulations using a soft-core potential and then with a short Langevin simulation using the full potential. Details on the contact map, structure clustering and domain analysis of the Pou5f1 and Sox2 simulations are provided in the SI.

## Results

### Modeling of the mESC Pou5f1 locus

To gain insight into the mechanisms that regulate chromatin structure *in vivo* at the sub-TAD scale, we applied our nucleosome-resolution computational model to the 50 kb Pou5f1 gene locus of mESCs, located at chr17:35 484.6–35 534.6 kb (mm10) [67]. In mESCs, the Pou5f1 gene actively expresses a transcription factor key to the maintenance of pluripotency [68]. This 50 kb locus alternates between high and low nucleosome acetylation [36], and the acetylated/nonacetylated intervals form sub-TAD self-interacting domains (10s of kilobases) on the experimental Micro-C contact map [69] (Fig. 3A and Supplementary Fig. S7). At this locus, contacts between distant acetylated enhancers and promoters are observed. The Ensembl database [70] identified the Pou5f1 promoter at chr17:35 506.0 kb (P1). The locus also contains the active promoter of the Tcf19 gene, which is colocalized with a small acetylation peak at chr17:35 516.8 kb (P2, +11 kb from P1). Groups of strong enhancers that contact the two promoters are located at chr17:35 485.6–35 486.1 kb (E1, −20 kb from P1) and chr17:35 502.1–35 502.7 kb (E2, −4 kb from P1). Our simulations account for the modulation of nucleosome–nucleosome interactions by several regulatory factors: nucleosome positions, histone tail acetylation, linker histone H1, and BRD4 association. Our key focus is understanding to what extent epigenetic-dependent nucleosome interactions can play a role in the domain-like 3D organization of the considered locus, and if so, whether they can partially explain the observed contact map (Fig. 3A). While cohesin is a key factor involved in the establishment of TADs, it is not explicitly modeled in our simulations as it is not the focus of our investigation, but we will comment on its potential role at the target loci.

The simulations were conducted in a spherical space to mimic an isotropic confined nucleus environment. The space was supplemented with short, nonspecific chromatin fibers to recreate the average DNA concentration in mESCs nuclei. One initial structure after short relaxation simulations is shown in Fig. 3B. Nucleosome positions, linker DNA lengths, distributions of canonical and acetylated nucleosomes, histone H1 and BRD4 were determined by incorporating experimental sequencing data [35, 36, 38] (Fig. 3C).

### Nucleosome interactions induce 10-kilobase scale compartmentalization

**Figure 4.**
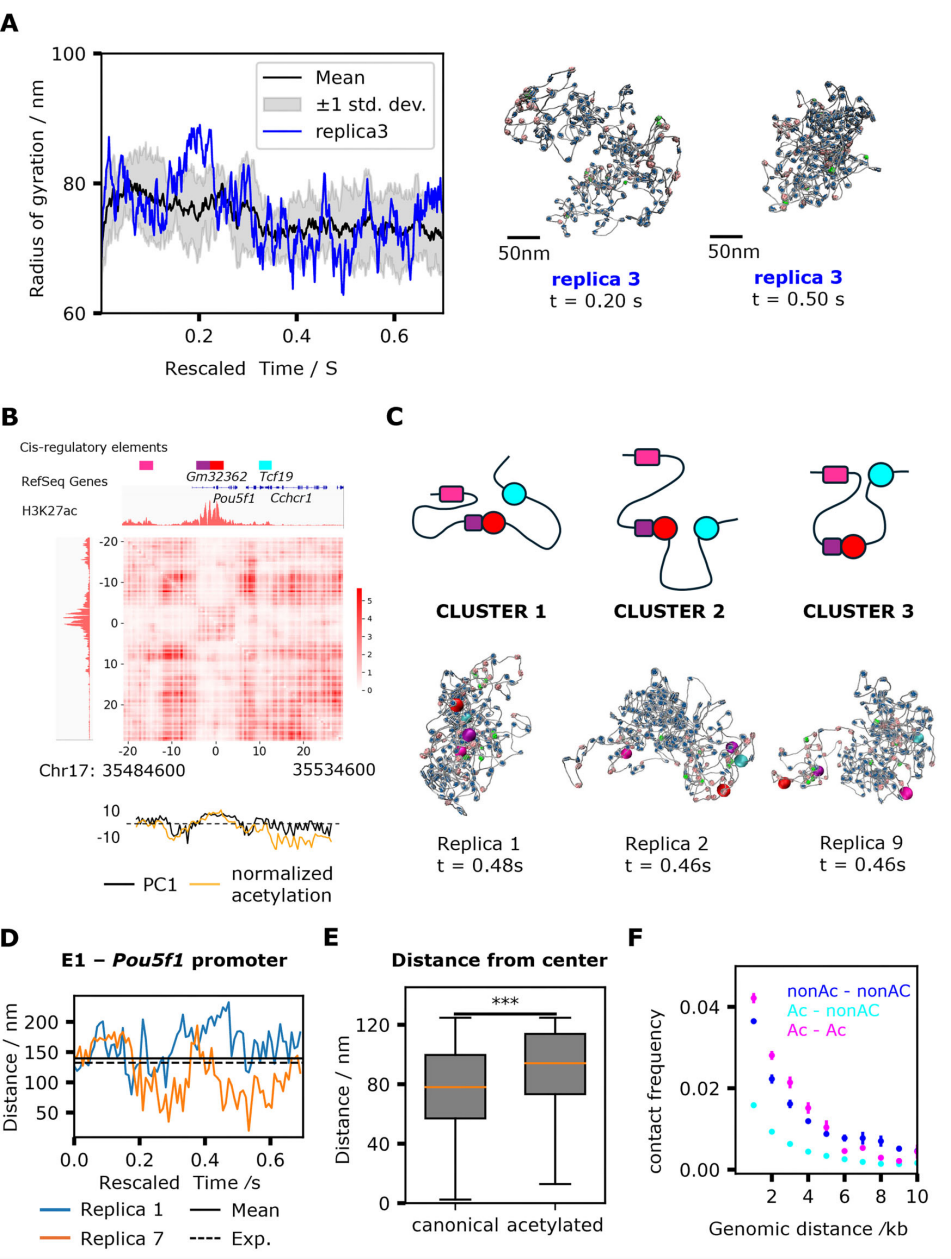
Nucleosome modification-mediated compartmentalization of Pou5f1 locus. (**A**) Mean radius of gyration (R_g_) over replicas and R_g_ in one representative replica of simulated models of the Pou5f1 locus. Representative snapshots of open (Replica 3, t = 0.20, R_g _= 88.5 nm) and compact (Replica 3, t = 0.50, R_g _= 63.1 nm) structures are shown. (**B**) Contact map calculated from Pou5f1 locus simulation trajectories, projection of genomic position on the first component from principal component analysis (PCA) of the contact map (PC1) compared with H3k27ac ChIP-seq signal. Data from t = 0.14s to t = 0.7s in nine simulation trajectories are used. Acetylation was normalized by taking the logarithm of signal in each bin divided by mean signal of all bins, multiplied by a factor for better visual comparison. (**C**) Illustration of regulatory element interactions in three major structure clusters (top). Representative structure snapshots from each cluster (bottom). Regulatory elements E1 (−20 kb), E2 (−4 kb), P1 (0 kb), P2 (13 kb) are colored magenta, purple, red, and cyan. (**D**) Trajectories of distance between enhancer E1 and the Pou5f1 promoter (P1) in two representative replicas. Horizontal line indicates mean distances calculated from nine replicas. (**E**) Distribution of canonical and acetylated nucleosomes’ distances from the center of the spherical simulation box. (**F**) Genomic distance-dependent contact frequency between a pair of canonical–canonical, canonical–acetylated, or acetylated–acetylated nucleosomes in the Pou5f1 locus. Data from t = 0.14s to t = 0.7s in nine simulation trajectories are used. Standard error was calculated using data from the nine replicas as independent samples. Pairs with distance above 10 kb were discarded because the sample size for acetylated pairs with genomic distance larger than 10 kb was very small.

We simulated 9 replicas of the Pou5f1 locus using different initial structures, each for 0.7 s (5 × 10^8^ timesteps). The simulation successfully captured dynamical fluctuations of chromatin structures (Supplementary Movie 1). The radii of gyration (R_g_) in different replicas had a mean value of 74.8 nm, with a relatively low standard deviation of 5.2 nm (Fig. 4A). The physical size of N-kb continuous regions within the Pou5f1 locus scaled as N^1/D^, with D = 2.9 (Supplementary Fig. S8A), consistent with experiments [71, 72] and the theory of crumpled globules [73]. The timeline of the radius of gyration over the replicas suggests convergence of the overall ensemble (Fig. 4A). While correlation between pairs of contact maps shows that the conformational ensembles sampled by different replicas become progressively more similar over time, convergence in individual trajectories is still out of reach (Supplementary Fig. S8B).

Even in the absence of cohesin, the simulated Pou5f1 locus clearly separated into three domains consistent with acetylation profile (Fig. 4B): the domain with intermediate level of acetylation (from −21 to −6 kb), the domain with high level of acetylation (−5 to −3 kb), and the domain with low level of acetylation (4–29 kb). Each domain had high self-contact frequency. PCA of the normalized contact map identifies 2 compartments, characterized by either high or low acetylation levels, with PC1 being correlated with acetylation (Fig. 4B, r = 0.55, *P *= 1.3 × 10^–10^). These results indicate that epigenetic modifications can play a significant role in the organization of the Pou5f1 locus.

We note that epigenetics alone cannot fully explain the experimental Pou5f1 contact map from Micro-C data (Supplementary Fig. S9). Quantitatively, PC1 calculated using normalized experimental Micro-C data showed visual correlation with simulation (Supplementary Fig. S9), but the correlation is not statistically significant (r = 0.08, *p *= 0.38). In the simulation contact map, the insulation scores at compartment boundaries had strong negative values, suggesting strong insulation between compartments, whereas the insulation scores calculated from experimental data were less pronounced (Supplementary Fig. S9). The compartmentalization coefficient in the simulation was 0.44, while 0.37 in experiment. This suggests that in Pou5f1 simulations the phase separation of nucleosomes depending on acetylation, H1, and BRD4 was the primary force facilitating sub-TAD compartmentalization, while loop extrusion, transcription elongation, and other factors acting *in vivo* but absent in our model could counteract this force. For example, in the experimental contact map a loop extrusion “stripe” brings the cohesin and CTCF binding sites near the Pou5f1 promoter in contact with the entire locus (Supplementary Fig. S7A), blending the boundaries between the two compartments, but this feature is absent in our simulations.

Direct communication between the two promoters and two enhancers mediated by nucleosome interactions was also observed in our simulations (Fig. 4C and D). The distance between the first enhancer and the Pou5f1 promoter (E1 and P1, genomic distance 20 kb) had a mean value of 140 ± 5 nm across replicas, comparable with the experimental value of 132 nm [74] (Fig. 4D). The standard deviation was 41 nm, while the experimental value was 84 nm. The additional heterogeneity *in vivo* may possibly be the result of heterogeneity at the level of epigenetic marks, which is absent in our model. K-means clustering of structures based on six pair-distances between regulatory elements (E1–E2, E1–P1, E1–P2, E2–P1, E2–P2, P1–P2) identified three major clusters of structures (Fig. 4C and Supplementary Fig. S10): in cluster 1, all regulatory elements interact in an overall compact chromatin structure; in cluster 2, E2, P1, and P2 interact, mediated by the clustering of acetylated nucleosomes; in cluster 3, E1, and P2 interact, mediated by the clustering of acetylated nucleosomes (Fig. 4C). The E1–P1 distance significantly fluctuated over the course of individual trajectories, with enhancer and promoter forming transient direct contacts on a sub-second timescale (Fig. 4D, trajectory of replica 7 shown in Supplementary Movie 2). However, the average E1–P1 distances varied across replicas, indicating the existence of different metastable conformational states that each replica can adopt. Enhancer–promoter contacts appear to be stabilized by the clustering of acetylated nucleosomes, in part associated with BRD4 (which mediates these interactions by bridging, representative snapshots shown in Fig. 4C).

To gain insights into the overall organization of the Pou5f1 locus, we calculated the distributions of distances of canonical and acetylated nucleosomes from the center of the spherical simulation space (Fig. 4E, snapshots in Supplementary Fig. S11). Interestingly, we find that canonical nucleosomes tend to occupy the core of the locus, while acetylated ones predominantly localize on the surface. This is in agreement with recent experiments and theoretical models [71, 75] suggesting that heterochromatin-like compact chromatin is located at the core of domains, while euchromatin-like active chromatin is located at the periphery, where the transcription machinery is recruited.

Analysis of the contact frequency between nucleosome pairs as a function of their genomic distance confirms the role of epigenetics in compartmentalization: pairs of nucleosomes with the same chemical modifications (canonical–canonical or acetylated–acetylated) showed higher contact frequency compared to pairs with different modifications (Fig. 4F).

### Chromatin forms highly dynamic domains

**Figure 5.**
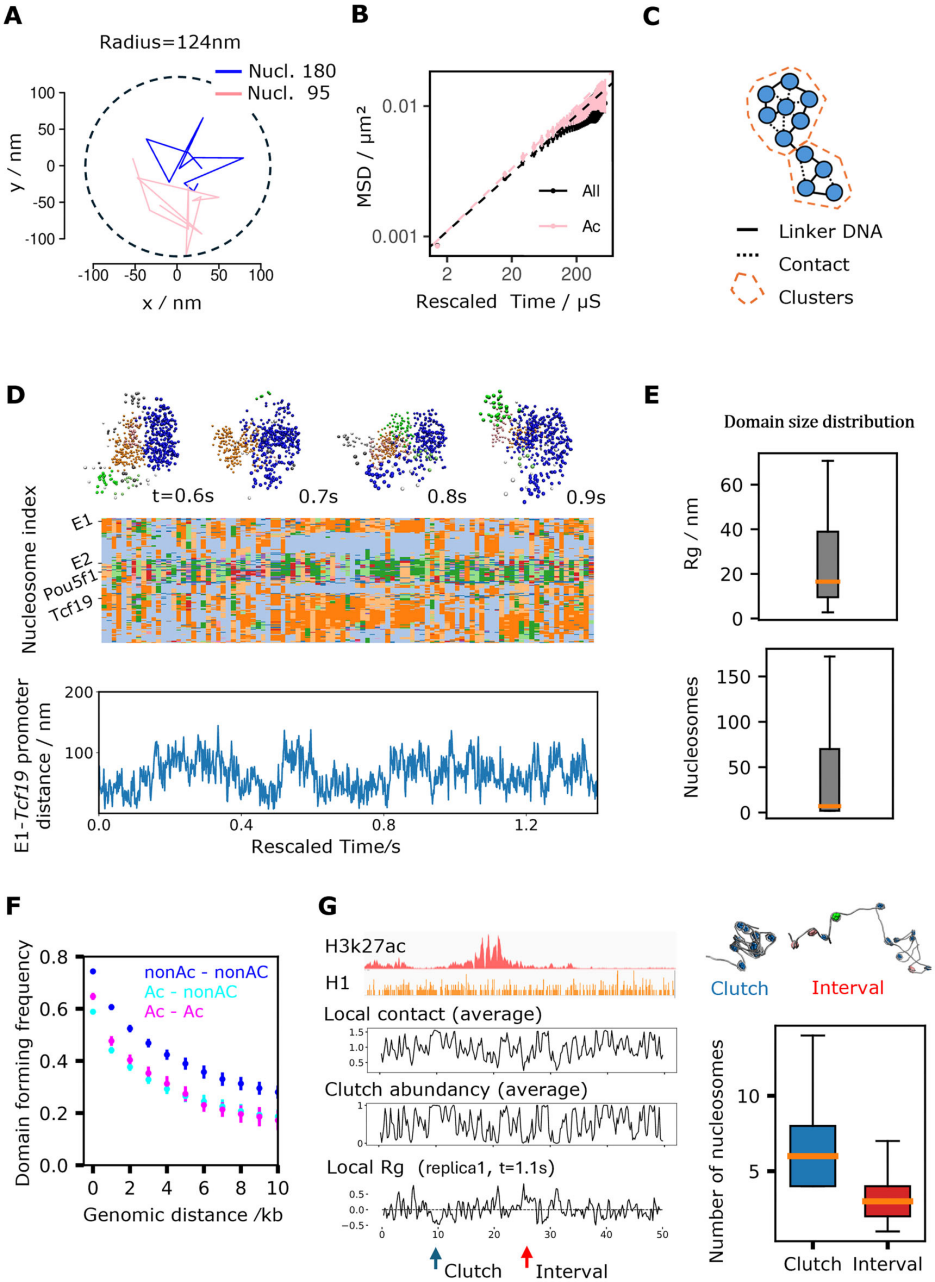
Multiscale dynamics of Pou5f1 locus. (**A**) Trajectories of the 180th nucleosome (canonical) and the 95th nucleosome (acetylated) from the extended simulation of the Pou5f1 locus. Coordinates were taken every 0.14s. (**B**) Mean squared displacement of all nucleosomes and acetylated nucleosomes in the Pou5f1 locus, as a function of lag time. Dashed lines are fittings to linear model. (**C**) Illustration of Infomap clustering applied to our chromatin system. Nucleosomes are the nodes in blue. (**D**) Representative snapshots of chromatin domains in the Pou5f1 locus (top). Each nucleosome is visualized as one bead. “Background” nonspecific chromatin fibers are included in the visualization. Time series of domains plotted every 0.014 s from the extended Pou5f1 locus simulation (center). Domains are indicated by different colors. Trajectory of the Enhancer1–Tcf19 Promoter distance (bottom). (**E**) Distribution of Infomap domain sizes in terms of radius of gyration (top) and number of nucleosomes (bottom). (**F**) Frequency of a pair of canonical–canonical, canonical–acetylated, acetylated–acetylated nucleosomes being in the same domain as a function of genomic distance. Standard error was calculated using data from nine replicas as independent samples. Data from t = 0.14 s to t = 0.7s were used to avoid the effects of initial structures. (**G**) Local contact frequency, clutch abundance (probability of each nucleosome being in a clutch), and four-nucleosomal R_g_ from one structure snapshot plotted on the Pou5f1 locus (left). Representative structures of one nucleosome clutch and one interval between clutches (right, top). Distribution of number of nucleosomes in clutches and intervals (right, bottom).

We next studied the dynamics of 10-kb-scale domains involved in compartmentalization. For this analysis, we extended one Pou5f1 simulation to 1 × 10^9^ timesteps (1.4 s). We find that despite forming well-defined compartments, single nucleosomes (both canonical and acetylated) diffuse over a vast space on our timescale of seconds, suggesting liquid-like, highly flexible chromatin structures (Fig. 5A).

The mean square displacement (MSD) of nucleosomes follows a power law relation MSD = K_α_τ^α^ at small-enough lag time τ, before a plateau due to confinement (Fig. 5B). α was 0.45 for the average over all nucleosomes, and 0.48 for acetylated nucleosomes, consistent with experiments [76] (α = 0.45 for all nucleosomes, α = 0.52 for nucleosomes in active early replication foci). Given the same exponent α, we could estimate the scale factor of 1.4 ns/timestep to map simulation times to real times by comparing the generalized diffusion coefficient K_α_ between simulations and experiments.

To study the Pou5f1 locus dynamics, we used the Infomap community detection algorithm [77] (Supplementary Information) to assign all nucleosomes in the simulation to domains according to their spatial proximity (Fig. 5C). This was done for every simulation frame to characterize how these domains change over time. Fig. 5D shows that while domains tend to be stable for about 0.4 s, chromatin is also dynamic, with several domain splitting and merging events observed over the course of our 1.4 second trajectory, with such events being correlated with the fluctuation of enhancer–promoter distance (Fig. 5D). Other, shorter simulation trajectories also show significant dynamic transformations of chromatin (Supplementary Fig. S12).

The radius of gyration R_g_ of the domains is highly variable (Fig. 5E), ranging from about 10 nm, on the order of individual nucleosomes and small nucleosome clutches [1, 2], up to 60 nm, comparable to the size of packing domains observed in experiments [3, 4].

We calculated the probability of a pair of nucleosomes being in the same domain as a function of their genomic distances (Fig. 5F). As for contact frequencies, nucleosomes with the same modification have a higher chance to associate into a domain, again indicating that epigenetics drives compartmentalization.

### Epigenetic-dependent nucleosomes clutches along the chromatin fiber

We investigated the organization of groups of consecutive interacting nucleosomes (clutches) based on the mean contact frequency along four-nucleosome sliding windows (Fig. 5F). When averaged over the simulation trajectory, the mean contact frequencies anticorrelated with acetylation level, as expected from their weaker interactions. When considering individual simulation frames, the R_g_ along four-nucleosome windows alternates between intervals of high and low values: we consider a continuous region in which R_g_ remains below average as one nucleosome clutch. Based on the analysis of clutches in the second half of the 1.4-s Pou5f1 trajectory, clutch sizes vary between 4 and 14 nucleosomes (with a mean of 6.9), which is consistent with experimental estimates [2]. Intervals between clutches vary between 1 and 19 nucleosomes, with a mean of 3.2. The probability of being part of a clutch in the trajectory was correlated with low acetylation and H1 binding (Fig. 5G). Underlying the formation of these clutches, we found stacking to be a key interaction contributing to chromatin compaction in our simulations. Stacking was defined based on a nucleosome–nucleosome distance cutoff of 7.5 nm and an angle between the two axes lower than 30 degrees. Stacking was found to make up about 20% of all nearest-neighbor interactions, but only about 3% for pairs of acetylated nucleosomes. Distance and angle distributions of nearest-neighbor interactions are shown in Supplementary Fig. S13 according to interaction type. Based on the angle distribution, stacking interactions appear more prominent compared to that observed in human T-lymphoblasts from cryo-electron tomography (cryo-ET) [78], but the difference could be due to the absence of *in vivo* factors that disrupt chromatin organization in our simulations.ee Long-range contacts are widespread: among stacked nearest neighbors, about 48% of them occur between nucleosomes separated by 10 or more other nucleosomes along the genome, while the remaining can be considered local contacts.

### Regulatory factors of nucleosome interactions are essential for chromatin hierarchical organization

**Figure 6.**
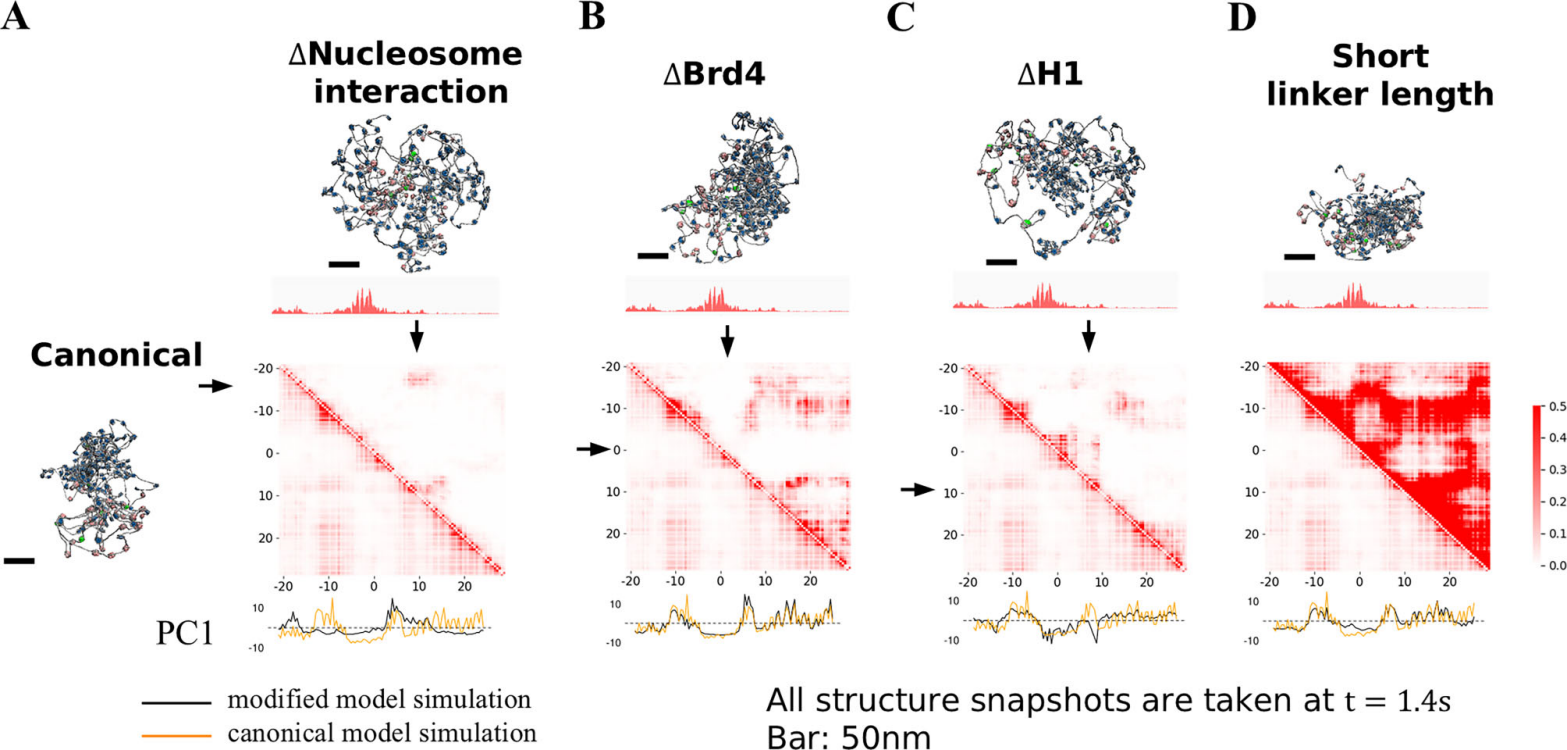
The impact of nucleosome interaction regulatory factors loss on chromatin compaction and compartmentalization. (**A–D**) Contact frequency maps and snapshots of the final structures of the modified Pou5f1 locus with the final structure of the canonical Pou5f1 locus as a reference. (**A**) Interactions involving canonical nucleosomes are reduced. (**B**) BRD4-associated nucleosomes are replaced by acetylated nucleosomes without BRD4. (**C**) Linker histone H1 is removed from the target Pou5f1 locus. (**D**) Compared to the canonical case, every linker length has been reduced by 1 DNA bead (10.5 bp), reducing the overall NRL. Below the contact maps we also show the projections of genomic loci on the first principal component from the canonical and the modified model simulations.

To assess the role of each individual nucleosome epigenetic factor for the 3D organization of the Pou5f1 locus, we performed 1.4-second simulations mimicking various experimental conditions using the following four models (Fig. 6): (i) Δnucleosome interaction: all nonbonded interaction strengths involving canonical nucleosomes are reduced to ε = 0.06 kcal/mol to mimic a widespread histone acetylation (although these nucleosomes will not experience enhanced interactions with BRD4-bound nucleosomes), (ii) ΔBRD4: BRD4-associated nucleosomes are replaced by acetylated nucleosomes without BRD4. (iii) ΔH1: linker histone H1 is removed from the target Pou5f1 locus. (iv) Short linker: compared to the canonical system, every single linker is reduced by 1 DNA bead (10.5 bp), reducing the overall NRL. The short chromatin fibers in the environment remained unmodified in these new simulations.

**Figure 7.**
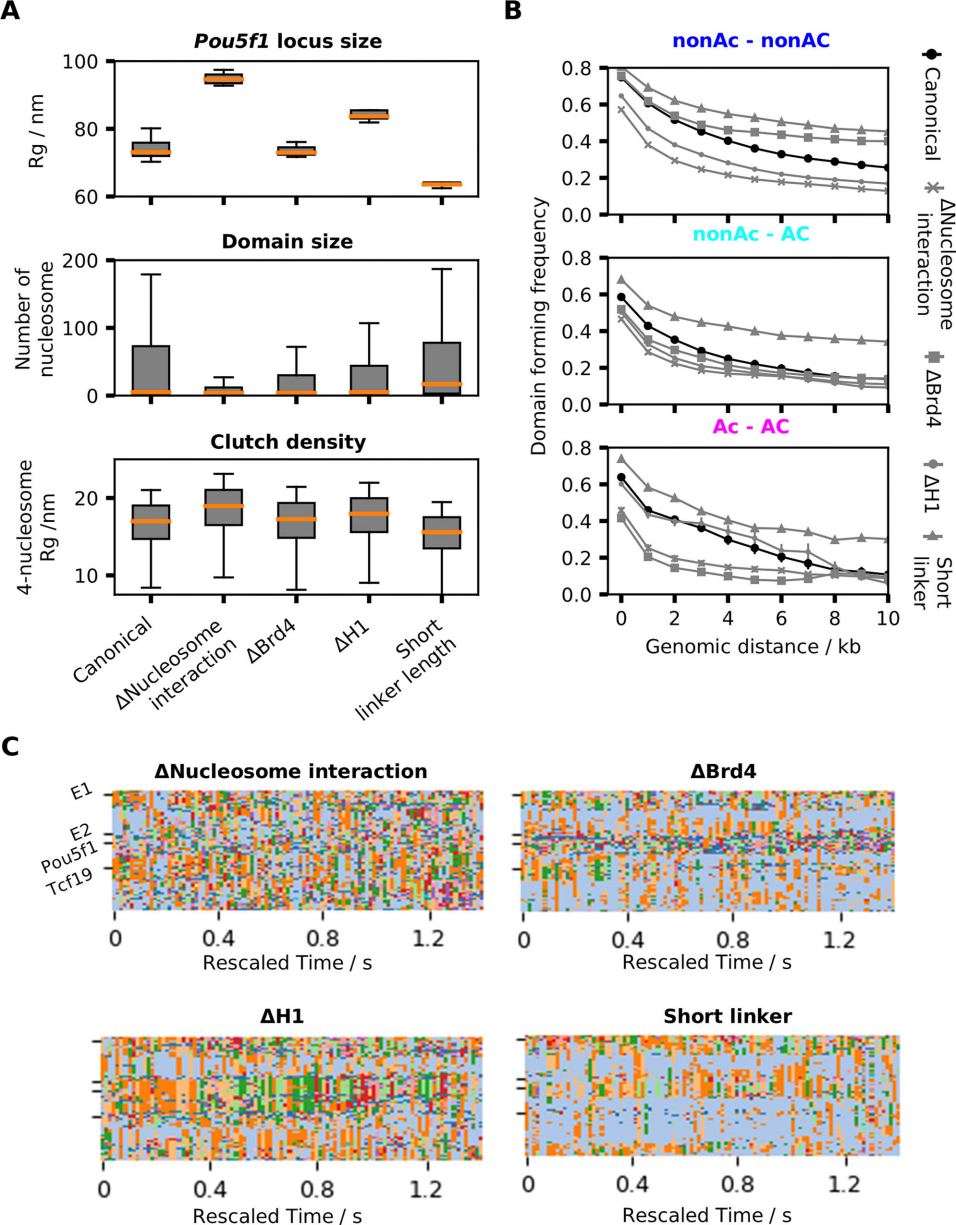
The impact of nucleosome interaction regulatory factors loss on chromatin compaction, domain formation and local chromatin fiber structures. (**A**) Distribution of size (R_g_) in the canonical and modified Pou5f1 locus simulations (top). Distribution of Infomap domain size (number of nucleosomes) in the canonical and modified Pou5f1 locus simulations (center). Distribution of R_g_ in regions identified as clutches in the canonical and modified Pou5f1 locus simulations (bottom). (**B**) Frequency of a pair of nonacetylated–nonacetylated (top), nonacetylated–acetylated (center), acetylated–acetylated (bottom) nucleosomes in the canonical and modified Pou5f1 locus simulations being in the same domain as a function of genomic distance. (**C**) Time series of domains from the modified simulations indicated by different color. Plotted every 0.02 s.

Reducing nucleosome interactions causes a reduction in the contact frequencies visible in the unnormalized contact map (Fig. 6A), an increase in the physical size of the locus (Fig. 7A), and significant changes in compartmentalization (Fig. 6A). Due to the intact BRD4-mediated bridging between some of the acetylated nucleosomes, enriched interactions between enhancer E1 and Pou5f1 promoter are still observed (arrows in Fig. 6A), and the two acetylated sites are categorized as the same compartment by PC1.

In the ΔBRD4 simulation, locus size (Fig. 7A) and compartments (Fig. 6B) remain largely unchanged compared to the unmodified model. However, the self-interacting domain at the −5 to 3 kb acetylation peak is lost (Fig. 6B), while E1–P1 and E2–P2 interactions are weaker. Repeating the same domain analysis as Fig. 5D, we found a decrease in association between acetylation peaks (Fig. 7C). This is also reflected in the probability of pairs of acetylated nucleosomes being in the same domain, which is lowest in the absence of BRD4 among the various conditions (Fig. 7B). These results confirm the key role of BRD4 for the formation of active chromatin domains.

In the absence of H1, we observe an increase in Pou5f1 locus size (Fig. 7A) and a shift in domain formation near the moderately acetylated region at 4–9 kb (Fig. 6C). Instead of interacting with the upstream low-acetylation region between two acetylation peaks, this region forms contacts with its neighboring acetylation peak, shifting from the canonical compartment to the acetylated compartment (Fig. 6C). This shift suggests a tug of war between different nucleosome interactions during compartmentalization: either mediated by H1 or by BRD4. The level of acetylation normally dictates whether one genomic region will be in a mostly acetylated compartment or a mostly nonacetylated compartment, but H1 depletion disrupts the balance of the two mechanisms, resulting in compartment shifts. H1 depletion further causes a decrease in the mean number of nucleosomes within Infomap domains from ∼46 to ∼33 (Fig. 7A). Clutches are also affected, with the mean value of 4-nucleosome R_g_ increasing to 17.7 nm from 16.7 nm (Fig. 7A).

Finally, reducing the overall NRL causes a significant compaction of the locus in terms of radius of gyration accompanied with more extensive long-range contacts, but the overall A/B compartment profile as defined by PC1 is unchanged compared to the canonical Pou5f1 simulation (Figs 6 and 7). Such behavior can be understood as originating from a reduction in the overall linker DNA charge and in the entropic cost of stacking interactions, which normally counteract the favorable nucleosome–nucleosome attraction. These observations are also in line with prior molecular simulations at nucleosome resolution [34].

### Exploring the structure and dynamics of the 120-kb Sox2 locus

**Figure 8.**
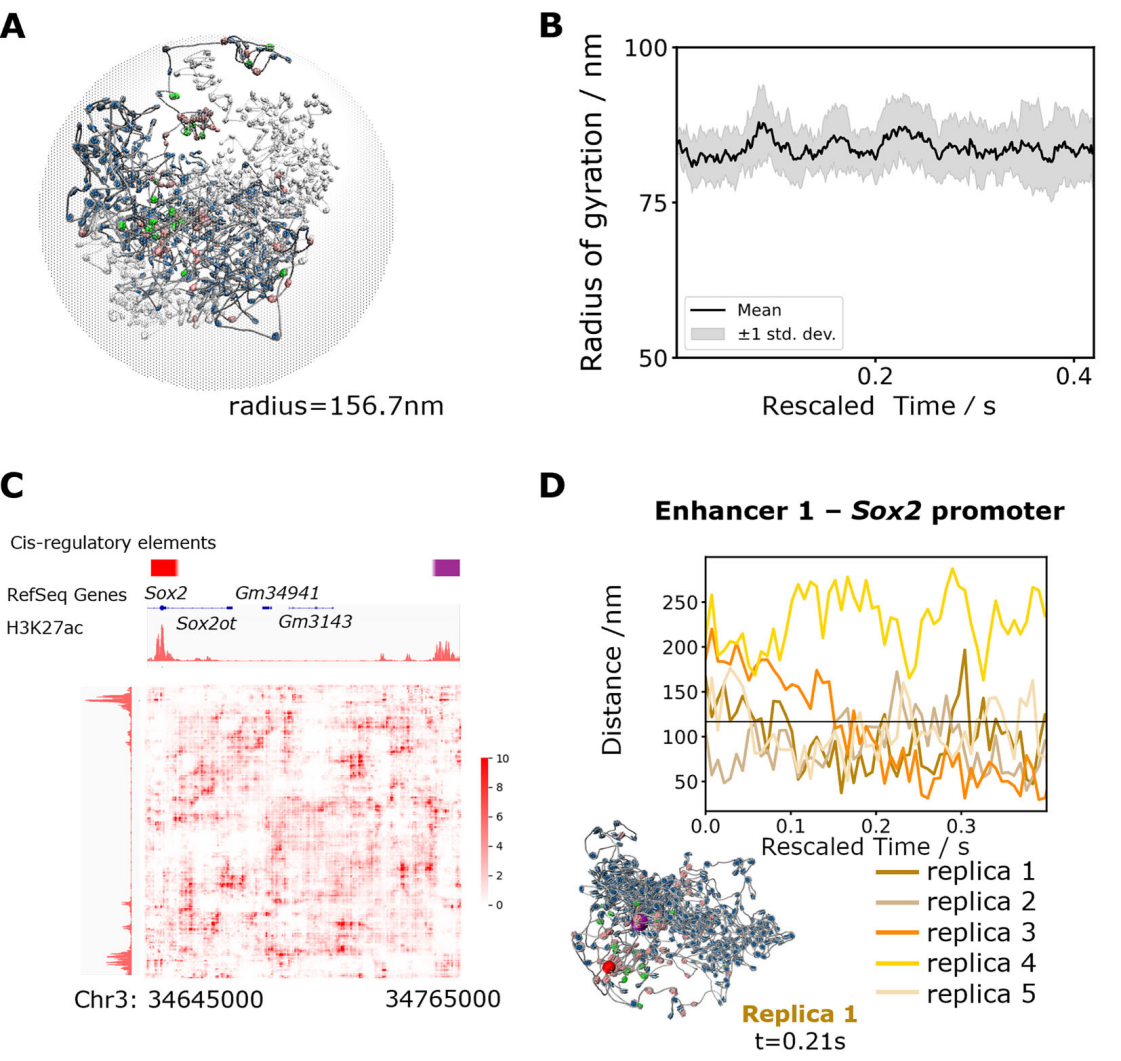
Simulation of the Sox2 locus. (**A**) Initial structure of the Sox2 locus after a short relaxation simulation. (**B**) Radius of gyration of five simulated replicas. (**C**) Contact map calculated from t = 0.2 s to t = 4.2 s of five replicas of Sox2 locus simulations. The Sox2 promoter and its strong enhancer are the acetylation peaks highlighted in red and purple, respectively. (**D**) Trajectories of the enhancer–promoter distances in five replica simulations as a function of rescaled time, with one representative snapshot of enhancer–promoter interaction (promoter in red, enhancer in purple).

To test the capability of our physics-based nucleosome-resolution model on larger systems, we constructed the model of a 120 kb region of mESCs, at chr3:34 645 kb–34 765 kb (mm10), containing the Sox2 gene, simply referred to as the Sox2 locus hereafter. Sox2 is also a transcription factor and pluripotency regulator expressed in mESCs [68]. Enriched contacts between two distal acetylated regions are observed in the experimental contact map (Supplementary Fig. S7B). The Sox2 promoter is located at chr3:34 650.4 kb. Gene expression is controlled by a strong enhancer at chr3:34 756.5 kb–34 761.5 kb, ∼109 kb downstream from the promoter [79]. While enriched contacts colocalize with cohesin binding at this locus, we wanted to ask whether nucleosome–nucleosome interactions could partially contribute to enhancer–promoter communication at this locus in addition to loop extrusion. Using a setup analogous to that of Pou5f1 (Fig. 8A), we simulated five independent replicas of the Sox2 locus for 0.42 s each (Supplementary Movie 3).

The mean R_g_ of the Sox2 replicas was 83.8 nm, with a standard deviation of 4.7 nm (Fig. 8B). R_g_ in each replica fluctuated around its mean value, showing convergence of this observable. Similar to the Pou5f1 locus, the size of Sox2 follows a power law with R_g_∝N^1/3^ (Supplementary Fig. S8A). Overall, the Sox2 locus appears more compact compared to Pou5f1, perhaps due to the comparatively higher amount of H1 in this region.

There is no significant convergence between contact maps calculated from independent replicas (Supplementary Fig. S8B), indicating that replicas sample distinct metastable basins. However, we do observe that in four out of five simulations the Sox2 promoter forms contacts with the 109 kb downstream enhancer after 0.15 s (Fig. 8D). Visual analysis of the structures suggests that these contacts are driven by interactions between acetylated nucleosomes mediated by BRD4.

## Discussion

In this study, we designed and parameterized a nucleosome-resolution chromatin model, named NICG, capturing the effects of key epigenetic factors that modulate nucleosome–nucleosome interactions: linker histone H1, histone tail acetylation, and BRD4 association. We applied this model to explore the structural dynamics of the 50-kb Pou5f1 and 120-kb Sox2 loci of mESCs, focusing on the potential role of epigenetic-dependent nucleosome interactions to govern the 3D organization of these loci *in vivo*.

Our model is notable for being computationally efficient and physics-based, being rigorously parametrized based on a large set of experimental data including chromatin sedimentation coefficients and validated on chromatin liquid–liquid phase separation. The sole use of point particles and simple interaction potentials allows it to be easily implemented using most MD software (in our applications, LAMMPS) [48] and to make use of high-performance computing solutions. For example, our Sox2 simulations included 200 kb of chromatin and could run at a speed of 18 ms/day on a 112-core cluster. This allows us to study not only the structural organization of large genomic loci but also significant structural changes that occur over the timescale of seconds.

Our in-depth study of the Pou5f1 locus of mESCs reveals that nucleosome–nucleosome interactions can play a significant role in the 3D organization of this gene. We find that the pattern of histone acetylation governs Pou5f1 folding, with contact maps showing one heterochromatin-like compartment enriched in canonical nucleosomes often associated with linker histone H1, and another compartment including active enhancers that is enriched in acetylated nucleosomes sometimes associated with BRD4. Our *in silico* mutation studies clarify the distinct roles of separate epigenetic factors: acetylation weakens nucleosome interactions to expand chromatin, linker histone H1 is the main driver of compaction, while BRD4 bridges acetylated nucleosomes to bring together distant regulatory regions. We note that while these are not unexpected features given our knowledge of these factors, the simulations using our physics-based model suggest that epigenetics is likely to have strong effects on the genome organization *in vivo*. This agrees with a recent experimental study showing that intrinsic nucleosome interactions, as measured by their tendency to form condensates, is sufficient to explain large-scale A/B compartmentalization [11]. Our work expands on this to further show that these effects are likely relevant for sub-TAD compartmentalization as well [10].

In vivo, epigenetic-driven nucleosome–nucleosome interactions likely act in concert with (or are antagonized by) other physical mechanisms such as cohesin loop extrusion [8]. The role of nucleosome interactions could potentially explain puzzling experimental observations showing that contacts between promoters and distant enhancers are often minimally affected when cohesin activity is perturbed [9]. According to our model, in the absence of loop extrusion enhancer–promoter communication could be rescued by interactions between acetylated nucleosomes bridged by BRD4, which can recover the phase separation of acetylated chromatin fibers *in vitro* [12].

Our simulations also offer insights into the functional organization of active loci such as Pou5f1. We find the typical organization of this locus consists of a heterochromatin-like core enriched in canonical nucleosomes surrounded by active acetylated regions that include enhancers and promoter, making these regions more accessible by the transcriptional machinery. This picture aligns with recent experimental and computational works [71, 75, 80]: our simulations suggest that this organization naturally arises from intrinsic nucleosome interactions. The local structure of the chromatin fiber is also functionally organized into compact clutches typically enriched in canonical nucleosomes and H1, and separated by intervals of acetylated nucleosomes. The literature contains a variety of terms describing different levels of chromatin domains, but our analysis highlights how domains exist on a continuum, from small clutches [1] to intermediate packing domains [3] to larger compartments [6, 10], and that nucleosome interactions alone are sufficient to give rise to such multiscale organization.

Underlying these domains, our simulations show that nearest-neighbor nucleosome stacking is prevalent among canonical nucleosomes but significantly reduced by acetylation (Supplementary Fig. S13). This physical mechanism aligns with recent cryo-ET data demonstrating that charge-neutralizing histone citrullination disrupts stacking to unfold native chromatin [81]. Ultimately, the modulation of these local contacts serves as a fundamental determinant of large-scale chromatin phase separation and material properties, a multiscale principle recently demonstrated for other structural variations, such as linker DNA length [82].

Finally, our long simulations reveal the liquid-like nature of chromatin organization: despite domains being relatively stable, we observe several domain splitting and merging events over the timescale of seconds. Furthermore, we could also observe large changes in the distance between enhancers and promoter, which within 1 s may go from being 200-nm apart to come as close as 50 nm, sufficient to accommodate direct contacts mediated by the RNA polymerase-mediator complex. This indicates that enhancer–promoter communication is transient and occurs over timescales significantly faster than that of transcriptional bursting, in agreement with experiments and theoretical modelling [83]. Our results also rationalize the surprising observation that gene activation state appears to be mostly uncoupled from the underlying chromatin structure [84].

Our nucleosome-resolution model offers a flexible platform to explore the physical basis of the interplay between chromatin organization and gene function. While the current work focused on epigenetic-dependent nucleosome interactions, future work should aim to simulate the dynamics of a gene locus *in vivo* including additional physical processes that likely play a role to functionally organize the 3D genome (e.g. cohesin loop extrusion and transcription factor condensation) [85]. Finally, our computationally lightweight model is ideally suited to be augmented with experimental biases (for instance from Micro-C data) [69] to implicitly model *in vivo* effects that cannot be directly captured by the physical model [23, 24].

## Supplementary Material

gkag535_Supplemental_Files

## Data Availability

Scripts, input files and trajectories for the reported NICG simulations have been uploaded to the Biological Structure Model Archive (BSMA) at the entry with DOI 10.51093/bsm-00092. Scripts and examples to setup NICG simulations are found at https://github.com/gbrandani/NICG_chromatin_model.
