# Supplementary material for: Physics-based nucleosome-resolution modeling of epigenetic-driven chromatin domain dynamics
Source: Nucleic Acids Res. 2026 May 26;54(10):gkag535. doi: 10.1093/nar/gkag535 (PMC13202176; doi:10.1093/nar/gkag535)
Supplement: gkag535_Supplemental_Files [file gkag535_supplemental_files.zip › movie_captions.docx]

**Supplementary Movie 1: *Pou5f1* dynamical fluctuations.** The movie illustrates the large-scale structural dynamics of the full *Pou5f1* system during the simulation.

**Supplementary Movie 2: Transient enhancer-promoter contacts.** The movie shows a representative simulation trajectory (Replica 7) of the *Pou5f1* locus, highlighting the formation of direct, transient contacts between the E1 enhancer and the *Pou5f1* promoter on a sub-second timescale.

**Supplementary Movie 3: *Sox2* dynamical fluctuations.** The movie illustrates the large-scale structural dynamics of the full *Sox2* system during the simulation.
