# Supplementary material for: Physics-based nucleosome-resolution modeling of epigenetic-driven chromatin domain dynamics
Source: Nucleic Acids Res. 2026 May 26;54(10):gkag535. doi: 10.1093/nar/gkag535 (PMC13202176; doi:10.1093/nar/gkag535)
Supplement: gkag535_Supplemental_Files [file gkag535_supplemental_files.zip › SI.pdf]

Physics-based nucleosome-resolution modeling of epigenetic-driven chromatin domain dynamics - Supplementary Methods

Details of model parametrization

Particle types are listed and described in Supplementary Table S1. Force field form and parameters are listed in Supplementary Table S2.

1. DNA persistence length simulation

1050 base pairs of B-type DNA were modeled as a polymer of 100 beads, connected by harmonic bonds Lennard-Jones interaction and electrostatic interaction was applied between beads (Supplementary Table S1). The angle potential was applied to neighboring bonds. Simulations were run at salt concentrations 2mM, 4mM, 10mM, 40mM, 150mM and 500mM, each for 10^9 steps.

DNA persistence length was calculated from the autocorrelation (1) $C(n) = \langle \cos \theta_{i,i+n} \rangle$ of all bond vectors separated by n bonds. $C(n)$ is fitted to an exponential decay:

$$C(n) = \exp\,(l/l_p)$$

Where distance $l = nl_b$, l_b is bond length, and l_p is the persistence length. l_p is calculated by fitting the $log\big(C(n)\big) \sim l$ relation to a linear model and taking the slope $b = l_p^{-1}$. The data for $l > 600$ nm is discarded to avoid finite length effect. The first 2×10^8 steps of the simulation under each condition are discarded to ensure simulation reaches equilibrium. The rest of the trajectory is divided into 4 parts, l_p is calculated from each part. DNA persistence length is calculated by block averaging as $\langle l_p \rangle$ and standard error was calculated as $\sigma(l_p)/\sqrt{4}$. The standard error of the fitted slope obtained from the least-squares regression was typically <0.1% of the persistence length values, negligible compared to the uncertainty arising from trajectory correlations. The same approach was applied for the investigation of the relation between the DNA persistence length and the coefficient K of the angle potential.

2. Nucleosome unwrapping simulation

Canonical nucleosome dimer was modeled as 2 canonical nucleosome core particles connected by 6 linker DNA beads (63 bp linker DNA). Equilibrium constant K_{eq} was defined as the ratio of the probability of unwrapped states to the probability of the fully wrapped state (2). The wrapped state was defined based on a threshold of 4 nm between DNA entry bead 22 and dyad DNA bead 15.

Acetylated nucleosome dimer is modeled as the same geometry, with Lennard-Jones interaction strength between nucleosome core particle beads and Morse bond strength between the entry/exit DNA bead and nucleosome core particle beads set to the acetylated values (Supplementary Table S1).

Nucleosome dimers are simulated 1×10^8 steps at 150mM salt concentration. We calculated the distance d between the exit DNA bead on the first nucleosome and the NCP-DNA bead 8

beads (approximately one turn of DNA wrapped on nucleosome) upstream, and defined structures where $d \leq d_0$ as wrapped states, structures where $d > d_0$ as unwrapped states. d_0 was set at 35nm. Each simulation trajectory was divided into 5 intervals, each 2×10^7 steps. K_{eq} was calculated in the last 4 intervals to ensure simulation reached equilibrium. Standard error was calculated as $\sigma(K_{eq})/\sqrt{4}$.

3. Potential of mean force for nucleosome stacking

To quantify the effective interaction between pairs of nucleosomes, either both canonical or both acetylated, we performed for each system a 10^9-steps simulation containing the 2 nucleosomes (without hanging linker DNA) inside a cubic box with 40 nm sides. We then computed the one-dimensional potential of mean force (PMF) as a function of the center–center separation, focusing specifically on stacked configurations (Figure S2). From the relative positions and orientations extracted from the trajectories, a stacking geometry was defined by imposing simultaneous constraints on two angles: one defined between the vector connecting the two nucleosome centers and the superhelical axis of the first nucleosome, and another angle between the two superhelical axes. In practice, both angles were required to be smaller than 30 degrees, ensuring face-to-face stacking. The PMF was obtained by Boltzmann inversion from the distance histogram of configurations satisfying these angular constraints. The PMF depth is slightly sensitive to definition of the stacking geometry, increasing by about 0.5 k_BT when the stacking angle cutoff is reduced from 30 to 20 degrees.

4. Nucleosome fiber sedimentation coefficients simulation

Sedimentation coefficient standardized to conditions corresponding to pure water at 20 °C and extrapolated to zero protein concentration, $s_{20,w}$, was used to quantify the compaction of chromatin fibers (3–6).

Canonical 12-nucleosome fiber was modeled as 12 canonical nucleosome core particles connected by 11 linkers with lengths 6, 6, 5, 6, 6, 5, 6, 6, 5, 6, 6 beads. The linker DNA length in the considered experiments is 207-147=60bp ~ 5.7 coarse-grained DNA beads (5,7). There was no overhanging linker DNA on the outer side of the end nucleosomes. A single fiber was simulated at 20mM, 40mM, 60mM, 100mM, 150mM, 200mM salt concentrations, for 2×10^8 steps under each condition.

Acetylated 12-nucleosome fiber was modeled with the same geometry as canonical 12-nucleosome fiber, with Lennard-Jones interaction strengths and Morse bond strengths set to the acetylated values (Supplementary Table S1). A single fiber is simulated at salt concentration 150mM for 2×10^8 steps.

Canonical 12-nucleosome fiber with linker histone H1 was modeled as the canonical 12-nucleosome array with H1 placed at the dyad site of every nucleosome. A single fiber was simulated at salt concentrations 10mM and 150mM, for 2×10^8 steps under each condition.

We explored the dependence of the sedimentation coefficient of 12-nucleosome canonical chromatin fibers on the nucleosome repeat length (NRL) by varying this between 168, 179, 189, 200, and 210 bp (corresponding to linker DNA lengths of 2, 3, 4, 5, and 6 coarse-grained beads,

respectively). Simulations were performed at a salt concentration of 150mM for 10^8 MD steps for each condition.

$s_{20,w}$ was calculated using the method described by Arya *et. al.* (8):

$$s_{20,w} = s_1\left(1 + \frac{2R}{N}\sum_{i}^{N}\sum_{j>i}^{N}\frac{1}{R_{ij}}\right)$$

Using the same value of s_1 ($s_{20,w}$ of one single nucleosome) and R (diameter of one nucleosome) as in previous computational studies (3). N is the number of nucleosomes and R_{ij} is the distance between two nucleosomes.

$s_{20,w}$ was calculated for each structure snapshot. The first 1×10^8 steps of the simulation under each condition were discarded to ensure simulation reaches equilibrium. Autoregressive integrated moving average (ARIMA) analysis is performed to the time series of $s_{20,w}$ to determine the mean value and its error. ARIMA analysis was done using the R package "forecast"(9,10).

5. Nucleosome fibers phase separation simulations

4-nucleosome fibers were constructed as 4 nucleosomes connected by 4 beads linkers (42 bp linker length). 90 copies of the same fiber were placed in a $100\text{nm} \times 100\text{nm} \times 1000\text{ nm}$ periodic boundary simulation box. Canonical 12-nucleosome fibers were constructed as 12 nucleosomes connected by 4 bead linkers. 45 copies of the same fiber were placed in a $100\text{nm} \times 100\text{nm} \times 1000\text{ nm}$ periodic boundary simulation box. Acetylated 12-nucleosome fibers were constructed as 12 acetylated nucleosomes connected by 4 bead linkers. 45 copies of the same fiber were placed in a $100\text{nm} \times 100\text{nm} \times 1000\text{ nm}$ periodic boundary simulation box. Acetylated 12-nucleosome fibers associated with BRD4 were modeled as 10 acetylated nucleosomes (not associated with BRD4) and 2 acetylated nucleosomes associated with BRD4, connected by 4 bead linkers. The BRD4-associated nucleosomes were placed at the second and the 11th nucleosomes. 45 copies of the same fiber were placed in a $100\text{nm} \times 100\text{nm} \times 1000\text{ nm}$ periodic boundary simulation box.

Simulation for each type of nucleosome fiber was run for 1×10^8 steps, simulation convergence was confirmed by calculating the total number of nucleosome contacts (figure S2. Contact was counted when the distance between two nucleosome centers was smaller than 110 nm). The first 2×10^7 steps of each trajectory were discarded to ensure simulation reached equilibrium.

The one-dimensional nucleosome concentration was calculated along the long axis of the simulation box, the regions with a concentration higher than $0.05\text{ nucleosome}/\text{Å}$ were assigned as the condense phase (Figure S2). The three-dimensional nucleosome concentration was calculated for each frame to calculate the mean concentration in the condense phase.

We used a BDR4 / acetylated nucleosome ratio (~0.17) close to the value in active gene loci. We obtain the ratio from the observed number of BRD4 molecules (9~15 molecules) at the Pou5f1 gene locus in mouse ES cells (mESCs) (11) and the estimated number of acetylated nucleosomes at the same locus (see section "Mapping of nucleosome position and nucleosome modifications ").

6. 96-nucleosome array simulation

A 96-nucleosome array was constructed as 96 nucleosomes connected by 95 linkers of 6 beads (63 bp linker length). The $(i \times 12 + 1)$ -th to the $((i + 1) \times 12)$ -th nucleosomes were acetylated nucleosomes, $i = 0, 2, 4, 6$. The $(j \times 12 + 1)$ -th to the $((j + 1) \times 12)$ -th nucleosomes were canonical nucleosomes, $j = 1, 3, 5, 7$. 10 replicas of the same array were simulated for 1×10^8 steps with different random seeds. The first half of all trajectories were discarded to ensure simulation reached equilibrium. Contact frequency was calculated by normalizing contacts counted in all trajectories to the total amount of structure frames. Contact was defined by the sigmoid function described in the section "Contact map data analysis" in Supplementary methods.

Mapping of nucleosome positions, nucleosome acetylation, linker histone H1 and multi-bromodomain protein BRD4

The center of nucleosome core particles (nucleosome dyads) were mapped to the genome using the chemical mapping data of nucleosomes (12) (GEO accession number: GSM2183909). Genomic coordinates in each target locus (Pou5f1 and Sox2) were sorted by their chemical mapping score. We assigned nucleosomes to the coordinate with the highest score that is at least 146 bp from other already assigned nucleosomes and repeat this process until no more nucleosomes can be assigned. Linker length was determined by subtracting 146 bp from the distance between two neighboring nucleosome dyads. In the case where linker DNA length couldn't be represented by an integer number of beads, rounding up was applied to ensure at least one linker bead presented between two nucleosome core particles.

Acetylated nucleosomes were assigned to nucleosomes according to their H3K27ac ChIP-seq signals in mESCs (13) (GSM3399478). The ChIP-seq signal S of one nucleosome was defined as the ChIP-seq signal integrated in the 146 bp interval centered at the nucleosome center position. The acetylation state of a nucleosome was decided statistically using a random number generator. If the random number $q \leq S/S_0$, the nucleosome was assigned as acetylated. The normalization factor S_0 was calibrated by randomly sampling 10000 nucleosomes from the entire genome using the unique peaks of nucleosome chemical mapping score from GSM2183909. S of sampled nucleosomes were calculated and S_0 was set at 100.0 so that 1% of sampled nucleosomes were assigned as acetylated, comparable with the values ranging from 1.2% to 7 % reported in different cell lines (14,15).

Linker histone H1 was added to nucleosome dyad sites according to their H1 ChIP-seq signals in mESCs (GSM1199586). The same approach to assigning acetylated nucleosomes is applied here, with the normalization factor set so that histone H1/nucleosome ratio in randomly sampled nucleosomes is 0.36, consistent with the values ranging from 0.36 to 0.46 reported in mESCs (16,17).

BRD4 associated nucleosomes were assigned according to BRD4 ChIP-seq signals (18) (GSM2319260). BRD4 was first mapped to nucleosomes using the same approach described above. From the mapped nucleosomes, only nucleosomes already assigned as acetylated nucleosomes were assigned as acetylated nucleosomes associated with BRD4 in the model of target loci. The normalization factor was set so that the number of BRD4 molecules was 10 in the

Pou5f1 gene locus, consistent with the finding that the number of BRD4 molecules at the Pou5f1 transcription foci are 9~15 (11).

Construction of Pou5f1 locus and Sox2 locus initial structures

The sizes of the confined simulation spaces were decided so that the total amount of chromatin in the two setups were 100 kbp for Pou5f1and 200 kbp for Sox2. Radius of the simulation spaces were set at 124.4 nm for the Pou5f1 locus and 156.7 nm for the Sox2 locus.

To setup the initial structures, replicas of even more coarse-grained backbone structures were first simulated using LAMMPS. Chains of beads were simulated in the spherical confined space. Segments between two neighboring beads represented 1kb chromatin. Beads were connected by harmonic bonds with no angle potential, and a Lennard-Jones potential with cutoff (only repulsive interactions) was applied between beads. The bead sizes (bond length and repulsive potential cut off) were set at 22.5 nm. Chains representing the Pou5f1 locus (50 kbp) and 4 short chromatin fibers (each 10 kb) were placed in the 124.4 nm sphere. Chains representing the Sox2 locus (120 kbp) and 4 short chromatin fibers (each 16 kbp) were placed in the156.7 nm sphere.

Nucleosome resolution models of target loci were constructed by placing nucleosomes one by one (Figure S4). The nucleosome core particle model in Figure 1A was copied, rotated and translated so that the entry DNA bead was placed at the start of the backbone (for the first nucleosome in the chain) or at the end of the linker of the previous nucleosome. The axis of nucleosome was rotated to be parallel to the backbone. Linker DNA at the downstream side of the nucleosome was generated by placing beads representing linker DNA on the line extended from the last NCP-DNA bead to the exit DNA bead, with 35Å distances between beads. The number of linker DNA beads was decided using the linker DNA length decided in the previous section.

The nucleosome resolution chromatin structure then goes through a soft-core potential simulation using LAMMPS. The Lennard-Jones interaction strength was set at 0 at the beginning of the simulation and linearly increased over the course of 100000 timesteps until it reached the target values in Supplementary Table S2. Harmonic bond and angle potentials were set as in Supplementary Table S2 and electrostatic interaction were not applied in this simulation. This simulation allowed automatic adjustment of the initial structure to avoid clashes between nucleosomes. The structure was further relaxed using a Langevin simulation with the full model (parameters as in Supplementary Table S2) for 1×10^7steps to produce the initial structures presented in Figure 3B.

Contact map data analysis

“Hi-C” like contact maps were generated by calculating the contact frequency between nucleosomes. Contact was determined by a sigmoid function:

$$c = \frac{1}{1 + \gamma^6}$$

Where $\gamma = (\frac{r - d_0}{r_0})$, r is the distance between the center of two nucleosomes, d_0 is the nucleosome diameter, 110 Å and r_0 is set as 55 Å. Nucleosomes are mapped into 500 base pair

bins. Contact frequency in each bin is normalized by distance-dependent average.

Compartments were called by calculating the principal component analysis (PCA) of normalized contact map. Compartmentalization coefficient is calculated as $\frac{C_{in}-C_{out}}{C_{in}+C_{out}}$, where C_{in} is the sum of contact frequencies between bins in the same compartment, and C_{out} is the sum of contact frequencies between bins in different compartments.

Insulation score of one bin was calculated as the sum of contact frequencies between upstream bins and downstream bins in a 20-bin window (10 kbp) centered at the bin of interest, normalized by the mean value of all windows.

Visualization of experimental Micro-C data

Micro-C data (19) (GSE130275) were exported using Juicebox (20). Bin size was set as 500 base pair and contact frequency in each bin was normalized using the observed/expected normalization option.

***Pou5f1* Structure clustering using K-means.**

Distances E1-E2, E1-P1, E1-P2, E2-P1, E2-P2, P1-P2 were calculated every frame from the t > 0.14s parts of all 9 replica simulations. K-means clustering was performed using the Python module pyEMMA (21) using different number of clusters k. Within-cluster-sum-of-squares (WCSS) was calculated for different k, and k=3 was chosen as the optimal number of clusters using the elbow criterion (Figure S7).

Domain identification using Infomap Clustering

Chromatin structures were converted into a network, with nucleosomes as nodes and nucleosome-nucleosome contacts as links. Contacts were identified using the same sigmoid function described in the section "Contact map data analysis" in Supplementary methods.

Clusters were optimized by minimizing the map equation (22). The map equation describes the conciseness of describing the trajectory of a random walk on the network by "movements between clusters" and "movements within clusters". A python implementation of the algorithm was used (23). The depth level parameter was set as 1, meaning no sub-clusters were calculated. The sizes of clusters were automatically optimized.

After cluster calling for each structure frame, names were assigned to clusters frame by frame. To keep cluster names consistent throughout the time series, the name of cluster A_i in frame i will be inherited by A_{i+1}, the cluster with the most common members with cluster A_i, in frame $i+1$.

References

1. Kahn PC. Defining the axis of a helix. Computers & Chemistry. 1989 Jan 1;13(3):185–9.

2. Li G, Widom J. Nucleosomes facilitate their own invasion. Nat Struct Mol Biol. 2004 Aug;11(8):763–9.

3. Lequieu J, Córdoba A, Moller J, De Pablo JJ. 1CPN: A coarse-grained multi-scale model of chromatin. The Journal of Chemical Physics. 2019 June 7;150(21):215102.

4. Shogren-Knaak M, Ishii H, Sun JM, Pazin MJ, Davie JR, Peterson CL. Histone H4-K16 Acetylation Controls Chromatin Structure and Protein Interactions. Science. 2006 Feb 10;311(5762):844–7.

5. Hansen JC, Ausio J, Stanik VH, Van Holde KE. Homogeneous reconstituted oligonucleosomes, evidence for salt-dependent folding in the absence of histone H1. Biochemistry. 1989 Nov 14;28(23):9129–36.

6. Wang X, He C, Moore SC, Ausió J. Effects of Histone Acetylation on the Solubility and Folding of the Chromatin Fiber*. Journal of Biological Chemistry. 2001 Apr 20;276(16):12764–8.

7. Grigoryev SA, Arya G, Correll S, Woodcock CL, Schlick T. Evidence for heteromorphic chromatin fibers from analysis of nucleosome interactions. Proceedings of the National Academy of Sciences. 2009 Aug 11;106(32):13317–22.

8. Arya G, Zhang Q, Schlick T. Flexible Histone Tails in a New Mesoscopic Oligonucleosome Model. Biophysical Journal. 2006 July 1;91(1):133–50.

9. R Core Team. R: The R Project for Statistical Computing [Internet]. Vienna, Austria.: R Foundation for Statistical Computing; 2021. Available from: https://www.r-project.org/

10. Hyndman RJ, Khandakar Y. Automatic Time Series Forecasting: The forecast Package for R. Journal of Statistical Software. 2008 July 29;27:1–22.

11. Li J, Dong A, Saydaminova K, Chang H, Wang G, Ochiai H, et al. Single-Molecule Nanoscopy Elucidates RNA Polymerase II Transcription at Single Genes in Live Cells. Cell. 2019 July 11;178(2):491-506.e28.

12. Voong LN, Xi L, Sebeson AC, Xiong B, Wang JP, Wang X. Insights into Nucleosome Organization in Mouse Embryonic Stem Cells through Chemical Mapping. Cell. 2016 Dec 1;167(6):1555-1570.e15.

13. Ramisch A, Heinrich V, Glaser LV, Fuchs A, Yang X, Benner P, et al. CRUP: a comprehensive framework to predict condition-specific regulatory units. Genome Biology. 2019 Nov 8;20(1):227.

14. Roh TY, Cuddapah S, Zhao K. Active chromatin domains are defined by acetylation islands revealed by genome-wide mapping. Genes Dev. 2005 Jan 3;19(5):542–52.

15. Litt MD, Simpson M, Recillas-Targa F, Prioleau M, Felsenfeld G. Transitions in histone acetylation reveal boundaries of three separately regulated neighboring loci. The EMBO

Journal. 2001 May;20(9):2224–35.

16. Cao K, Lailler N, Zhang Y, Kumar A, Uppal K, Liu Z, et al. High-Resolution Mapping of H1 Linker Histone Variants in Embryonic Stem Cells. PLOS Genetics. 2013 Apr 25;9(4):e1003417.

17. Zhang Y, Liu Z, Medrzycki M, Cao K, Fan Y. Reduction of Hox Gene Expression by Histone H1 Depletion. PLOS ONE. 2012 June 11;7(6):e38829.

18. Aldiri I, Xu B, Wang L, Chen X, Hiler D, Griffiths L, et al. The Dynamic Epigenetic Landscape of the Retina During Development, Reprogramming, and Tumorigenesis. Neuron. 2017 May 3;94(3):550-568.e10.

19. Hsieh THS, Cattoglio C, Slobodyanyuk E, Hansen AS, Rando OJ, Tjian R, et al. Resolving the 3D Landscape of Transcription-Linked Mammalian Chromatin Folding. Molecular Cell. 2020 May 7;78(3):539-553.e8.

20. Durand NC, Robinson JT, Shamim MS, Machol I, Mesirov JP, Lander ES, et al. Juicebox Provides a Visualization System for Hi-C Contact Maps with Unlimited Zoom. Cell Syst. 2016 July;3(1):99–101.

21. Scherer MK, Trendelkamp-Schroer B, Paul F, Pérez-Hernández G, Hoffmann M, Plattner N, et al. PyEMMA 2: A Software Package for Estimation, Validation, and Analysis of Markov Models. J Chem Theory Comput. 2015 Nov 10;11(11):5525–42.

22. Rosvall M, Bergstrom CT. Maps of random walks on complex networks reveal community structure. Proceedings of the National Academy of Sciences. 2008 Jan 29;105(4):1118–23.

23. Edler D, Holmgren A, Rosvall M. The MapEquation software package [Internet]. 2024. Available from: https://mapequation.org

24. Grant CE, Bailey TL, Noble WS. FIMO: scanning for occurrences of a given motif. Bioinformatics. 2011 Apr 1;27(7):1017–8.

25. Correll SJ, Schubert MH, Grigoryev SA. Short nucleosome repeats impose rotational modulations on chromatin fibre folding. The EMBO journal. 2012; 31(10): 2416.

Supplementary Tables

Bead type	Name	Description
1	DLN	10.5 bp linker DNA
2	DNC	10.5 bp nucleosome core particle DNA not at the entry/exit site
3	DEX	10.5 bp nucleosome core particle DNA at the entry/exit site
4	H2A	Histone H2A in a canonical nucleosome
5	H2B	Histone H2B in a canonical nucleosome
6	H3	Histone H3 in a canonical nucleosome
7	H4	Histone H4 in a canonical nucleosome
8	DAN	10.5 bp nucleosome core particle DNA in an acetylated nucleosome, not at the entry/exit site
9	DAE	10.5 bp nucleosome core particle DNA in an acetylated nucleosome, at the entry/exit site
10	A2A	Histone H2A in an acetylated nucleosome
11	A2B	Histone H2B in an acetylated nucleosome
12	A3	Histone H3 in an acetylated nucleosome
13	A4	Histone H4 in an acetylated nucleosome
14	DBN	10.5 bp nucleosome core particle DNA in an acetylated nucleosome associated to a BRD4 molecule, not at the entry/exit site
15	DBE	10.5 bp nucleosome core particle DNA in an acetylated nucleosome associated to a BRD4 molecule, at the entry/exit site
16	B2A	Histone H2A in an acetylated nucleosome bound to a BRD4 molecule
17	B2B	Histone H2B in an acetylated nucleosome bound to a BRD4 molecule
18	B3	Histone H3 in an acetylated nucleosome bound to a BRD4 molecule
19	B4	Histone H4 in an acetylated nucleosome bound to a BRD4 molecule
20	H1	Linker Histone H1

Supplementary Table S1. Names and descriptions of bead types used in the nucleosome resolution chromatin model. Colors correspond to non-bonded interaction groups consistent with Figure 1 B-C in main text.

**Harmonic bonds** $E = k(r - r_0)^2$		
$k = 0.50$		r_0= native value
**Morse bonds** $E = D\left[1 - e^{-\alpha(r-r_0)}\right]^2$		
$D_0 = 2.20$<br>$D_{ac} = 1.80$	$\alpha = 0.3$	r_0= native value
**Angle potentials** $E = K[1 + \cos(\theta)]$		
$K = 8.8$		
**Lennard-Jones interactions** $E = 4\varepsilon\left[\left(\frac{\sigma}{r}\right)^{12} - \left(\frac{\sigma}{r}\right)^{6}\right], r < r_c$		
$\varepsilon_0 = 0.115$<br>$\varepsilon_{ac} = 0.060$<br>$\varepsilon_h = 0.083$<br>$\varepsilon_B = 0.180$<br>$\varepsilon_{H1} = 0.700$	$\sigma = 35.0$	$r_c = 79.538$
**Debye-Hückel interactions** $E = C\frac{q_i q_j}{\epsilon r}\exp(-kr), r < r_c$		
$\epsilon = 80$	$k = 0.325\sqrt{I}$	$r_c = 10/k$

Supplementary Table S2. Pair interaction parameters used in the coarse-grained chromatin model. Distance units are Å, Energy units are $\mathrm{kcal \cdot mol^{-1}}$. r_0 for each pair of beads in the nucleosome core particle are set as in our reference model (see main text). r_0 for each pair of linker DNA beads are set at 35.0Å. For Debye-Hückel interactions, $k = \sqrt{\frac{I}{\epsilon k_B T}}$ is the inverse of Debye length (unit: $Å^{-1}$). I is the ionic strength (unit: mol/L). In the Lennard-Jones energy constants ε, the suffix indicates the interaction parameter between beads of unmodified nucleosomes (0), acetylated nucleosomes (ac), mixed unmodified-acetylated nucleosome interactions (h), BRD4-bound (and acetylated) nucleosomes (B), and linker histone (H1). In the Morse bonds, D_{ac} is the parameter for all acetylated nucleosomes, including BRD4-bound.

Supplementary Figures

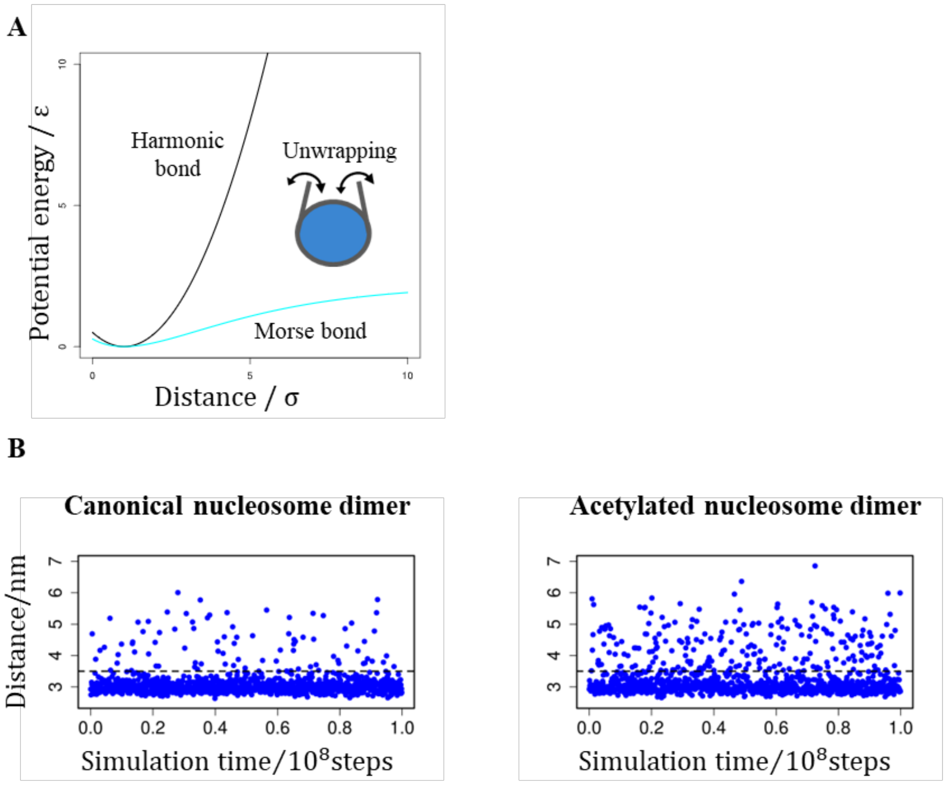

Figure S1. A. Comparison between the energy landscape of harmonic bonds and morse bonds. **B.** Simulation trajectory of the distance between the exit DNA bead and nucleosome core particle in a nucleosome dimer. Dashed line indicates the cutoff for unwrapped state, 3.5nm.

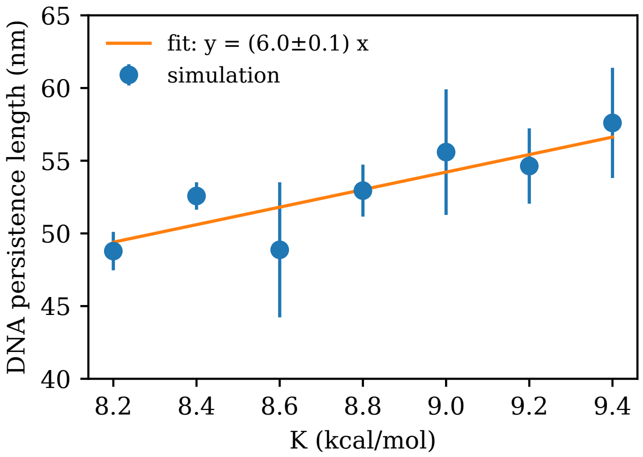

Figure S2. DNA persistence length vs stiffness coefficient K of the DNA angle potential, showing a linear fit with zero intercept consistent with the worm-like chain model.

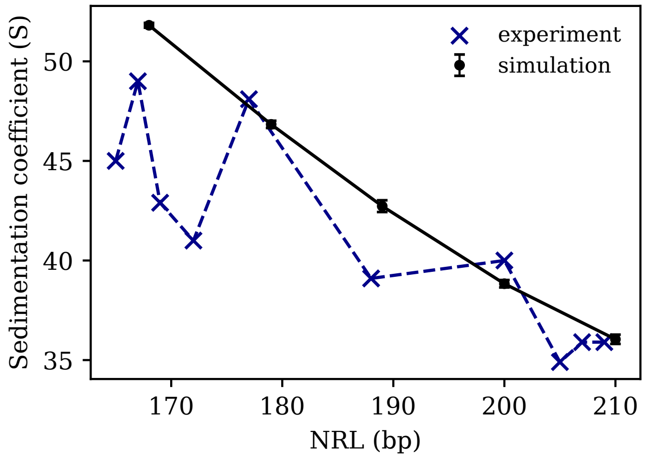

Figure S3. Sedimentation coefficients of 12-nucleosome chromatin fibers as a function of nucleosome repeat length (NRL): experiments (crosses) (25) vs simulations (circles). Simulation error bars represent the standard error of the mean estimated from time-correlated sedimentation trajectories.

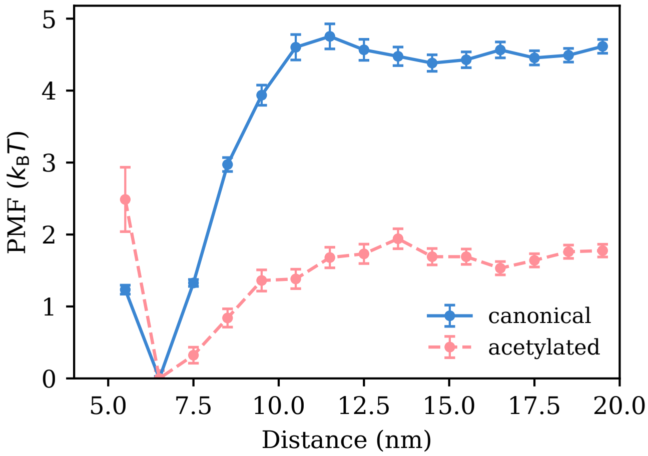

Figure S4. Potential of mean force (PMF) for nucleosome stacking as a function of the center-center distance for canonical (blue, solid) and acetylated (pink, dashed) nucleosomes. The PMF was computed from the distance distribution of configurations satisfying stacking constraints.

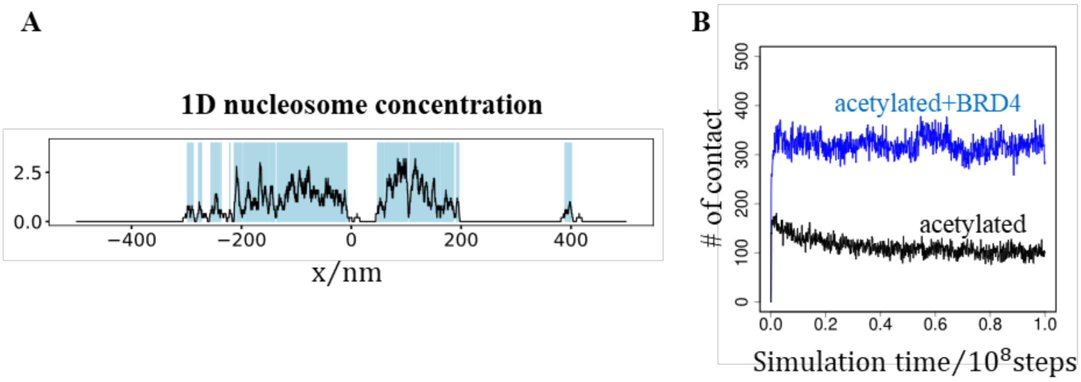

Figure S5. A. One dimensional nucleosome concentration at t=10^8 timesteps in the simulation of acetylated and BRD4 associated 12-nucleosome fibers. The unit of concentration is $Å^{-1}$. Regions highlighted in cyan are classified as condensates. **B.** Sum of nucleosome contacts in simulations of 12-nucleosome fibers.

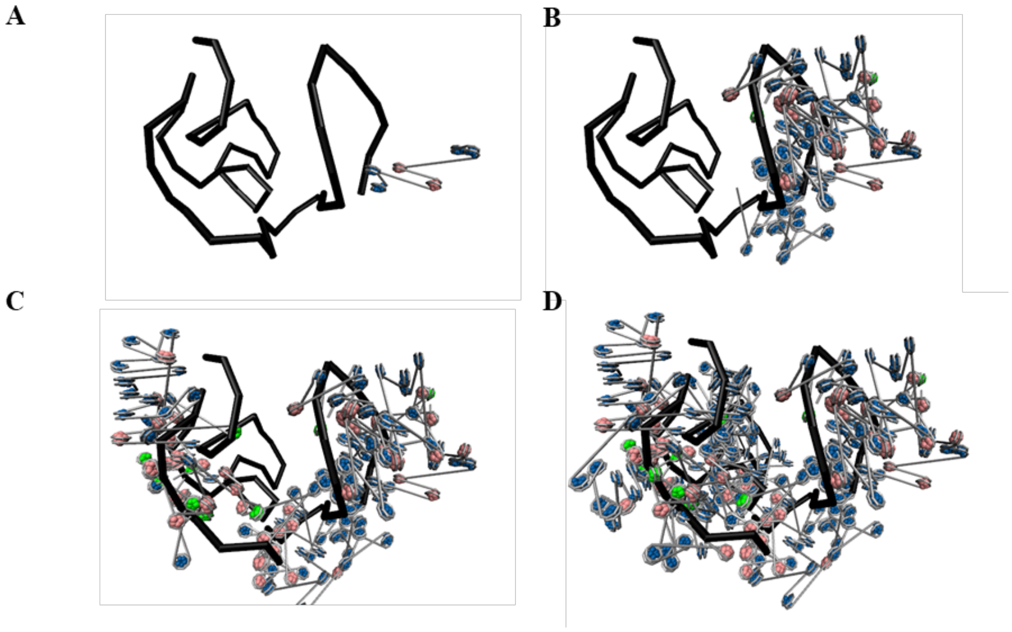

Figure S6. Illustration of the back mapping process placing nucleosome resolution chromatin model following the backbone model (black line), going from panel A to B, C, and D as the fiber grows.

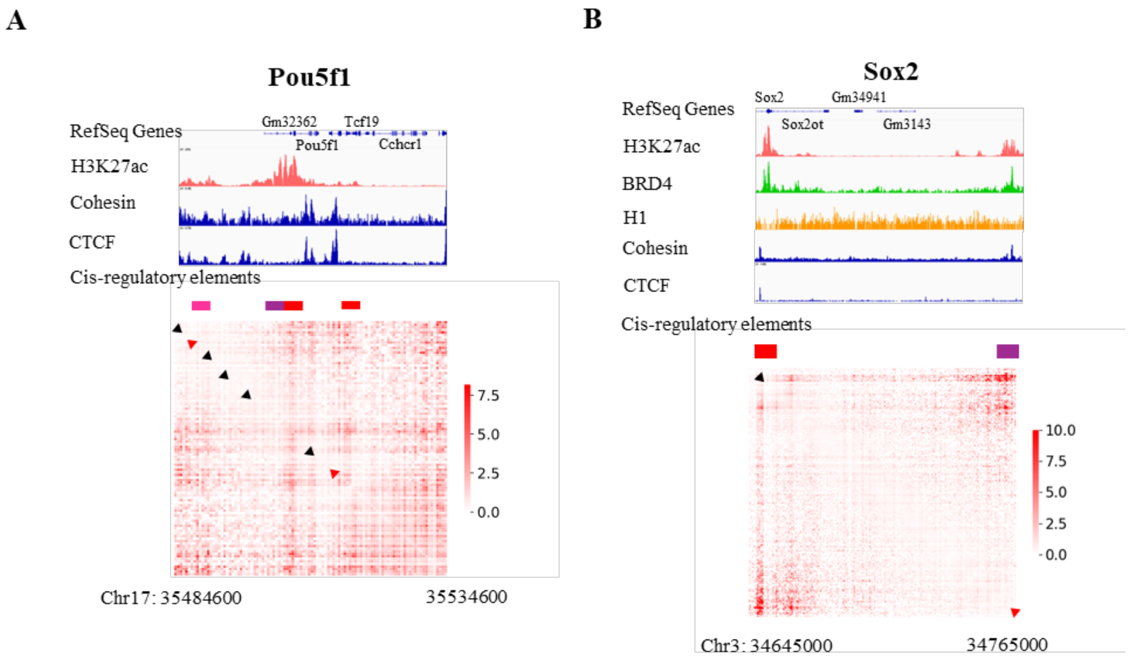

Figure S7. Normalized micro-C contact frequency data compared with ChIP-seq data for the Pou5f1 (A) and Sox2 (B) loci. On the contact map, we also show the CTCF binding motifs scanned using FIMO (24). Motifs located on the positive strand and negative strand are colored black and red, respectively, with arrows indicating the inside of loops if loop extrusion stops at the corresponding site.

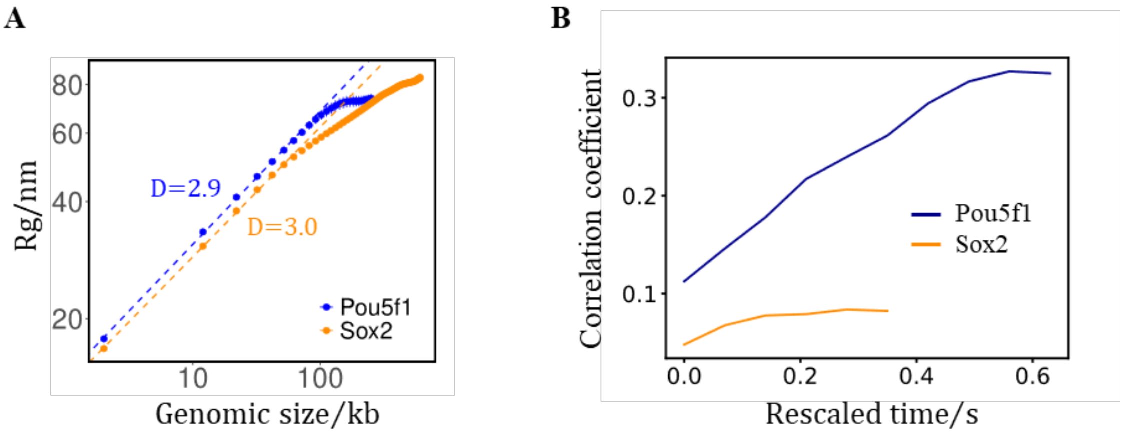

Figure S8. A. Radius of gyration – genomic length relations of continuous regions taken from the Sox2 and Pou5f1 loci. The x and y scales are logarithmic. The plot indicates the polymer fractal dimensions based on power-law fits $R_g \propto N^{1/D}$. **B.** Mean of Pearson Correlation coefficients between normalized pair-contact frequencies calculated from time $0 \sim \tau$ of any pair of replicas.

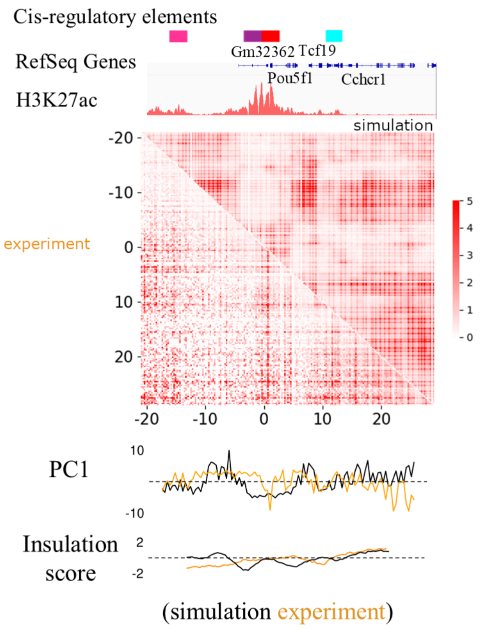

Figure S9. Comparison between simulation (top right) and experimental (bottom left) contact maps calculated from time $0.14s\sim0.7s$ from 9 *Pou5f1* locus simulation replicas. Below we also report PC1 of principal component analysis and insulation score from simulations compared with PC1 and insulation score calculated from the experimental contact map. The experimental contact map is normalized using the Knight-Ruiz method in Juicebox, then divided by 10 so that it's comparable with simulation data. The simulation data is normalized by genomic distance (divide by the mean contact frequency of all pairs with the same genomic distance).

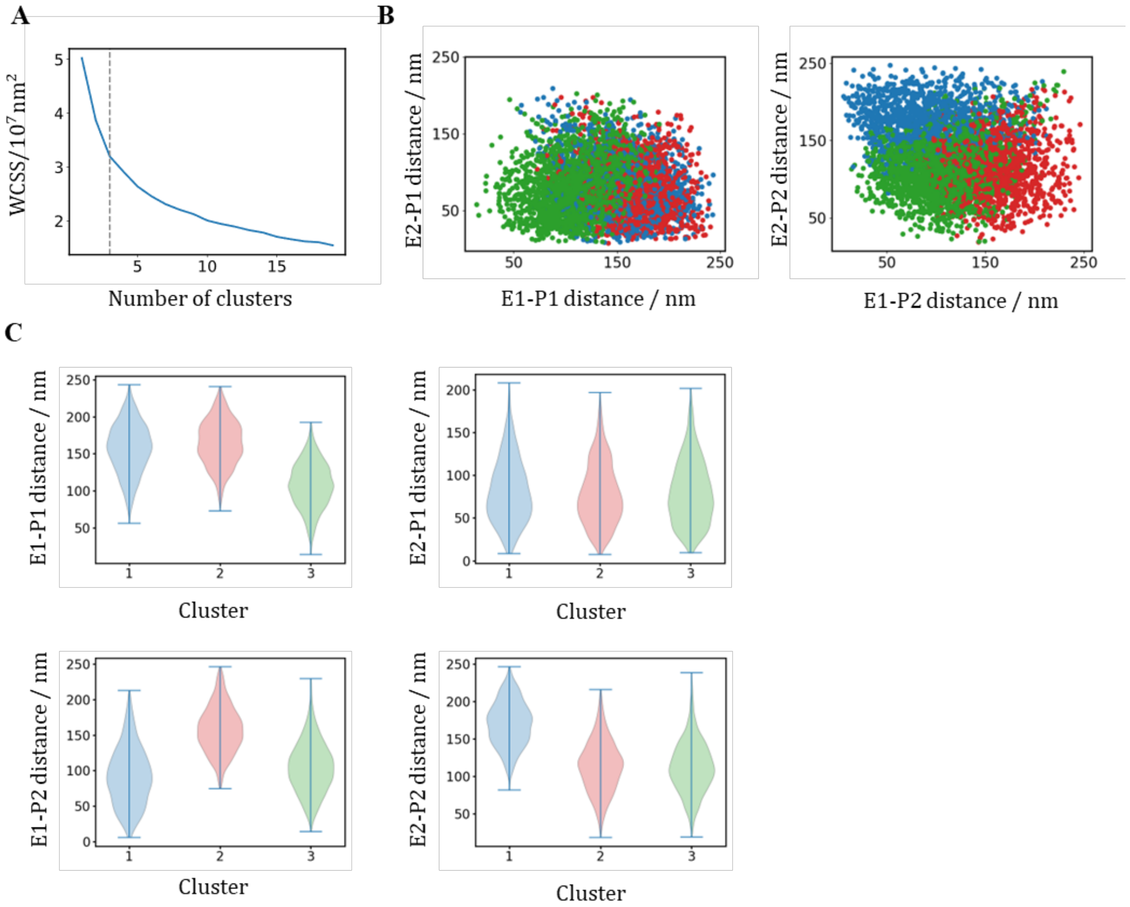

Figure S10. K-means clustering of simulated structures described by regulatory elements pair distances. **A.** Within-cluster-sum-of-squares (WCSS) as a function of number of clusters k used in K-means clustering. Dashed line indicates the chosen "elbow" point (k=3) **B.** Scatter plots of enhancer-promoter distances in 3 clusters. **C.** Violin plots of enhancer-promoter distances in 3 clusters.

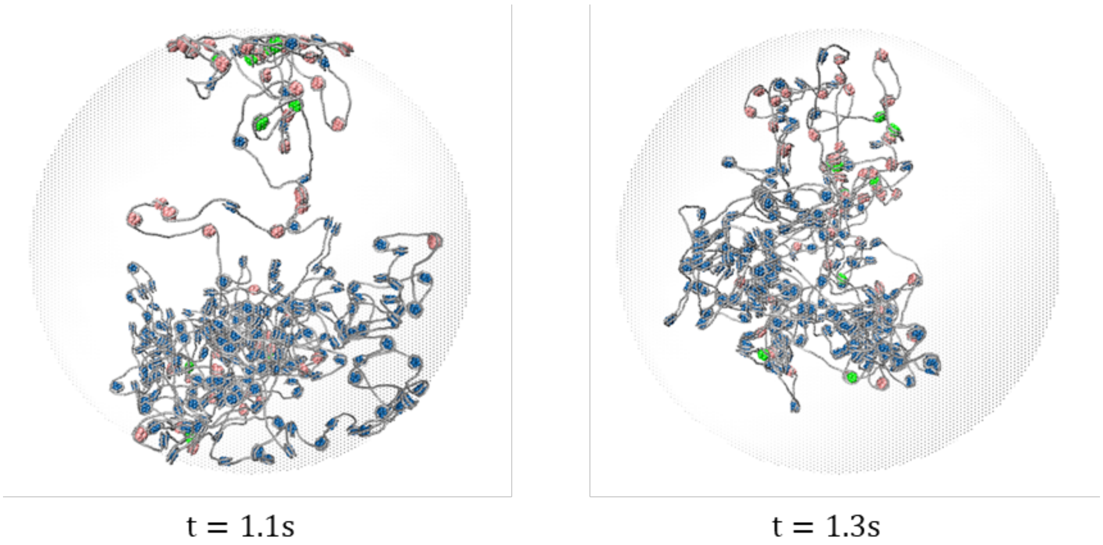

Figure S11. Representative structure snapshots from the extended (1.4s) simulation of Pou5f1 locus, with acetylated nucleosomes located on the periphery of the structures.

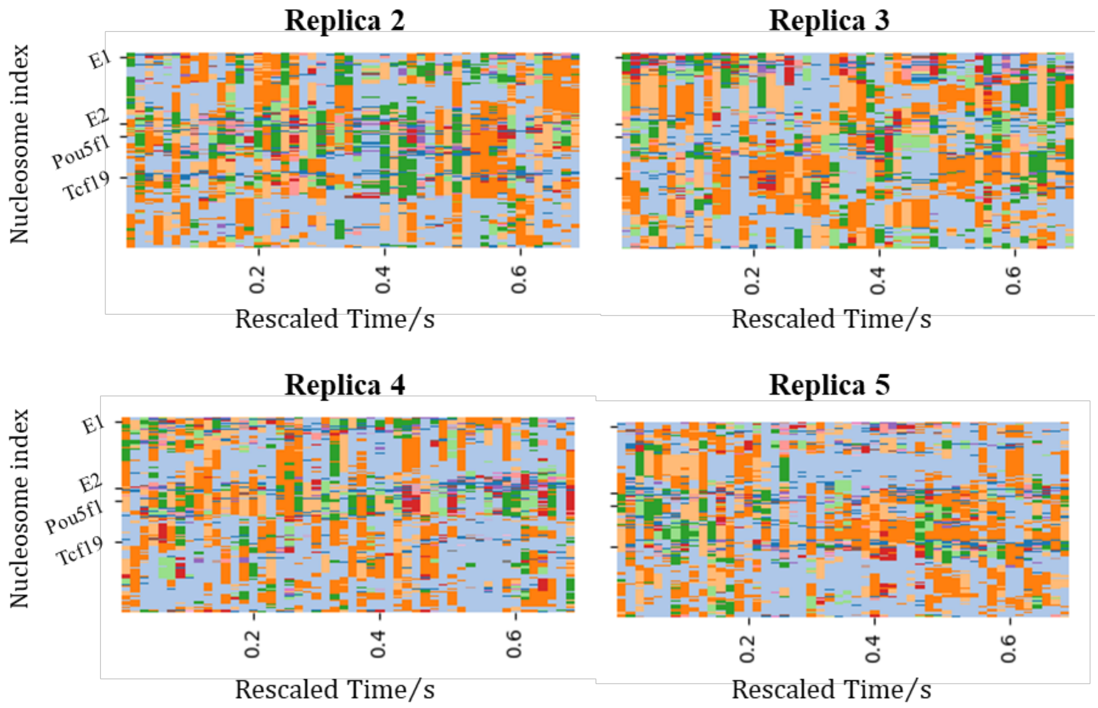

Figure S12. Example time series of domains identified from the $0.7s$ Pou5f1 locus simulation trajectories. Domains are indicated by different colors. Calculated every 0.014s.

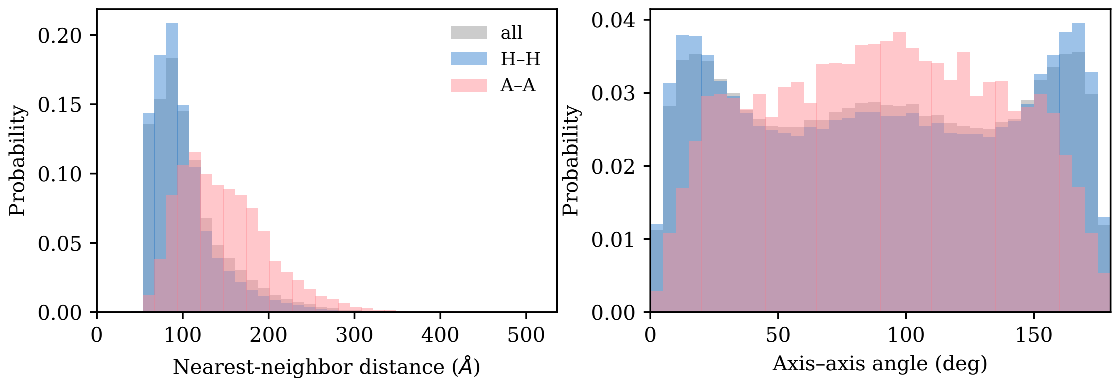

Figure S13. Nearest-neighbor nucleosome interactions in the Pou5f1 locus, showing the probability distributions of center–center distance (left) and angle between the superhelical axes (right) of neighboring nucleosomes. Distributions were independently computed for all nucleosome interactions pairs (all, in grey), limited to pairs of unmodified histones (H-H, in blue), and limited to acetylated histones (A-A, in pink). For this analysis we used the entire first Pou5f1 simulation trajectory.
